# Supplementary material for: Human brain changes after first psilocybin use
Source: Nat Commun. 2026 May 5;17:3977. doi: 10.1038/s41467-026-71962-3 (PMC13144397; doi:10.1038/s41467-026-71962-3)
Supplement: Supplementary file 1 — Supplementary Information [file 41467_2026_71962_MOESM1_ESM.pdf]

Human brain changes after first psilocybin use

SUPPLEMENTARY MATERIAL

Contents

1. Background

1.1. Table S1. Reference table for (to our knowledge) all previous psychedelic studies involving sub or post-acute neuroimaging or behavioural outcomes.

2. Study design and demographics.

2.1. Recruitment, screening, and eligibility criteria.
2.2. Figure S1. Study timeline of interventions.
2.3. Table S2. Schedule of interventions.
2.4. Table S3. Demographic data.

3. EEG outcomes

3.1. Table S4. EEG modeling of complexity and power changes in frequency bins and interaction between Dose and Timepoint.
3.2. Figure S2. Sensor-level EEG maps for LZc and power in frequency-bins.
3.3. Figure S3. Single subject changes in LZc and alpha power.
3.4 Additional controls pertaining the results of the data-driven analysis of LZc.

4. fMRI outcomes

4.1. Figure S4. fMRI BOLD responses to emotional faces (all face types) and all time points. Data drawn from 25mg vs 1mg cluster.
4.2. Figure S5. Response to faces with single subject values shown.
4.3. Amygdala (ROI) response to emotional faces.
4.4. Figure S6. Left amygdala BOLD response to emotional faces of various types.
4.5. Figure S7. Right amygdala BOLD responses to emotional faces.
4.6. Table S5. Amygdala psychophysiological interaction analysis: One-month post 1mg vs one-month post-25mg.
4.7 Figure S8. Amygdala psychophysiological interaction analysis: One-month post 1mg vs one-month post-25mg.
4.8. Figure S9. Bilateral amygdala BOLD RSFC analysis on key contrast.
4.9. Figure S10. Single subject data. Bilateral amygdala BOLD RSFC.
4.10. Figure S11. Whole-brain naïve ANOVA across all timepoints.
4.11. Validation analysis: in-scanner anxiety: amygdala RSFC analysis.
4.12. Resting State Networks (RSN) – Within and Between Networks RSFC.
4.13. Figure S12. Modularity with single subject values shown.
4.14. An overview of fMRI results

5. DTI outcomes

5.1. Figure S13. DTI: tracts where changes were observed.
5.2. Figure S14. Axial diffusivity values without free-water correction. Left hemisphere.
5.3. Figure S15. Axial diffusivity values with single subject datapoints shown.
5.4. Figure S16. Fractional Anisotropy (FA) with free-water correction.

5.5. Free-water correction values for axial diffusivity.

6. Psychological outcomes
6.1. Figure S17. IDED task phase 1-9.
6.2. Figure S18. IDED task: Cognitive flexibility (more granular) findings.
6.3. Figure S19. EDS errors. Single subject data.
6.4. Table S6. IDED task errors per criterion.
6.5. Figure S20. Adapted version of 'Persisting Effects Questionnaire' of Griffiths et al. 2006.

7. Sample size
7.1. Table S7. Sample size included in analyses per metric.

8. References

1. Background

1.1 Table S1. Summary of relevant research.

Reference and URL link	Brief description
Roseman L, Demetriou L, Wall MB, et al. (2018) Increased amygdala responses to emotional faces after psilocybin for treatment-resistant depression. *Neuropharmacology* 142: 263-269<br>https://doi.org/10.1016/j.neuropharm.2017.12.041	Increased amygdala responses to emotional faces after psilocybin for treatment-resistant depression (this group's work)
Mertens LJ, Wall MB, Roseman L, et al. (2020) Therapeutic mechanisms of psilocybin: Changes in amygdala and prefrontal functional connectivity during emotional processing after psilocybin for treatment-resistant depression. *Journal of Psychopharmacology* 34(2): 167-180.<br>https://doi.org/10.1177/0269881119895520	Decreased amygdala functional connectivity 1 day after psilocybin during a task in patients with depression (this group's work)
Stroud JB, Freeman TP, Leech R, et al. (2018) Psilocybin with psychological support improves emotional face recognition in treatment-resistant depression. *Psychopharmacology (Berl)* 235(2): 459-466.<br>https://doi.org/10.1007/s00213-017-4754-y	Enhancements of emotional processing 1 day after psilocybin in patients with depression (this group's work)
Doss MK, Považan M, Rosenberg MD, et al. (2021) Psilocybin therapy increases cognitive and neural flexibility in patients with major depressive disorder. *Translational Psychiatry* 11(1): 574.<br>https://doi.org/10.1038/s41398-021-01706-y	Enhanced cognitive flexibility 1 week after psilocybin in patients with depression
Barrett FS, Doss MK, Sepeda ND, et al. (2020) Emotions and brain function are altered up to one month after a single high dose of psilocybin. *Scientific Reports* 10(1): 2214.<br>https://doi.org/10.1038/s41598-020-59282-y	Decreased amygdala activation 1 week after psilocybin and no changes in within- and between-network connectivity in a healthy population (like the current study)
Smigielski L, Scheidegger M, Kometer M, et al. (2019) Psilocybin-assisted mindfulness training modulates self-consciousness and brain default mode network connectivity with lasting effects. *Neuroimage* 196: 207-215.<br>https://doi.org/10.1016/j.neuroimage.2019.04.009	fMRI 2 days after psilocybin
McCulloch DEW, Madsen MK, Stenbæk DS, et al. (2021) Lasting effects of a single psilocybin dose on resting-state functional connectivity in healthy individuals. *Journal of Psychopharmacology* 36(1): 74-84.<br>https://doi.org/10.1177/02698811211026454	fMRI 1 week and 3 months after psilocybin
Mason NL, Kuypers KPC, Reckweg JT, et al. (2021) Spontaneous and deliberate creative cognition during and after psilocybin exposure. *Translational Psychiatry* 11(1): 209.	fMRI acutely but also cognitive testing 1 week after psilocybin

https://doi.org/10.1038/s41398-021-01335-5	
Mason NL, Mischler E, Uthaug MV, et al. (2019) Sub-Acute Effects of Psilocybin on Empathy, Creative Thinking, and Subjective Well-Being. *Journal of Psychoactive Drugs* 51(2): 123-134.<br><br>https://doi.org/10.1080/02791072.2019.1580804	Cognitive testing 1 day and 1 week after psilocybin
Smigielski L, Kometer M, Scheidegger M, et al. (2019) Characterization and prediction of acute and sustained response to psychedelic psilocybin in a mindfulness group retreat. *Scientific Reports* 9(1): 14914.<br><br>https://doi.org/10.1038/s41598-019-50612-3	Predicting the sustained response of psilocybin
Sampedro F, de la Fuente Revenga M, Valle M, et al. (2017) Assessing the Psychedelic "After-Glow" in Ayahuasca Users: Post-Acute Neurometabolic and Functional Connectivity Changes Are Associated with Enhanced Mindfulness Capacities. *International Journal of Neuropsychopharmacology* 20(9): 698-711.<br><br>https://doi.org/10.1093/ijnp/pyx036	fMRI 1 day after ayahuasca
Pasquini L, Palhano-Fontes F and Araujo DB (2020) Subacute effects of the psychedelic ayahuasca on the salience and default mode networks. *Journal of Psychopharmacology* 34(6): 623-635.<br><br>https://doi.org/10.1177/0269881120909409	fMRI1 day after ayahuasca
Uthaug MV, van Oorsouw K, Kuypers KPC, et al. (2018) Sub-acute and long-term effects of ayahuasca on affect and cognitive thinking style and their association with ego dissolution. *Psychopharmacology (Berl)* 235(10): 2979-2989.<br><br>https://doi.org/10.1007/s00213-018-4988-3	Cognitive testing 1 day and 4 weeks after ayahuasca
Kiraga MK, Mason NL, Uthaug MV, et al. (2021) Persisting Effects of Ayahuasca on Empathy, Creative Thinking, Decentering, Personality, and Well-Being. *Frontiers in Pharmacology* 12:721537.<br><br>https://doi.org/10.3389/fphar.2021.721537	Cognitive testing 1 day and 1 week after ayahuasca
Bouso JC, Palhano-Fontes F, Rodriguez-Fornells A, et al. (2015) Long-term use of psychedelic drugs is associated with differences in brain structure and personality in humans. *European Neuropsychopharmacology* 25(4): 483-492.<br><br>https://doi.org/10.1016/j.euroneuro.2015.01.008	Another study, albeit observational in nature, that reported structural (grey matter) changes from a psychedelic i.e., ayahuasca use
Murphy-Beiner A and Soar K (2020) Ayahuasca's 'afterglow': improved mindfulness and cognitive flexibility in ayahuasca drinkers. *Psychopharmacology (Berl)* 237(4): 1161-1169.<br><br>https://doi.org/10.1007/s00213-019-05445-3	Enhanced cognitive flexibility 1 day after ayahuasca in an ayahuasca using population

Halpern JH, Sherwood AR, Hudson JI, et al. (2005) Psychological and cognitive effects of long-term peyote use among Native Americans. *Biological Psychiatry* 58(8): 624-631.<br><br>https://doi.org/10.1016/j.biopsych.2005.06.038	No effect on cognitive flexibility approximately 1 month after mescaline in a mescaline-using population
Wießner I, Olivieri R, Falchi M, et al. (2022) LSD, afterglow and hangover: Increased episodic memory and verbal fluency, decreased cognitive flexibility. *European Neuropsychopharmacology* 58: 7-19.<br><br>https://doi.org/10.1016/j.euroneuro.2022.01.114	Impaired cognitive flexibility 1 day after LSD
Ramos LR, Fernandes O Jr, Sanchez TA. (2025) Resilience and Brain Changes in Long-Term Ayahuasca Users: Insights From Psychometric and fMRI Pattern Recognition. *Journal of Magnetic Resonance Imaging.* EPub ahead of print.<br><br>https://doi.org/10.1002/jmri.70063	Altered emotional brain reactivity and increased psychological resilience in long-term ayahuasca users.
Zhu X, Zhang C, Hellerstein D, et al. (2025) Single-dose psilocybin alters resting state functional networks in patients with body dysmorphic disorder. *Psychedelics (N Y)* 1: 25-31.<br><br>https://doi.org/10.61373/pp024r.0028	fMRI 1 day after psilocybin.
Pagni BA, Petridis PD, Podrebarac SK, et al. (2024) Psilocybin-induced changes in neural reactivity to alcohol and emotional cues in patients with alcohol use disorder: an fMRI pilot study. *Scientific Reports* 14: 3159.<br><br>https://doi.org/10.1038/s41598-024-52967-8	fMRI 2 days after psilocybin.
Madsen MK, Petersen AS, Stenbaek DS, et al. (2024) CCH attack frequency reduction after psilocybin correlates with hypothalamic functional connectivity. *Headache* 64:55-67.<br><br>https://doi.org/10.1111/head.14656	fMRI 1 week after psilocybin.
Shukuroglou M, Roseman L, Wall M, et al. (2023) Changes in music-evoked emotion and ventral striatal functional connectivity after psilocybin therapy for depression. *Journal of Psychopharmacology* 37:70-79.<br><br>https://doi.org/10.1177/02698811221125354	fMRI 1 day after psilocybin.
Reiche S, Hirschfeld T, Gröticke AL, et al. (2025). Sporadic use of classic psychedelics and neuropsychological performance: A cross-sectional analysis. *Progress in Neuropsychopharmacology & Biological Psychiatry* 138:111353.<br><br>https://doi.org/10.1016/j.pnpbp.2025.111353	Increased cognitive flexibility in users of classical psychedelics.
Krabbe A, Sikka P, Jylkkä J. Acceptance as a possible link between past psychedelic experiences and psychological flexibility. *Scientific Reports* 14:24253.<br><br>https://doi.org/10.1038/s41598-024-75595-8	Quality and depth of the psychedelic experience linked to psychological flexibility, particularly Acceptance, and overall well-being.

2. Study design and demographics

2.1. Recruitment, screening, and eligibility criteria

Participants (*N*=28) were recruited via an online advertisement on the Centre for Psychedelic Research website. All participants initiated contact to gain their place on the study. After an initial telephone screen, participants were formally screened in the National Institute for Health Research/Wellcome Trust Imperial Clinical Research Facility (ICRF) to determine eligibility. Participants were emailed the participant information sheet outlining the nature, purpose and risks of the study in sufficient time for them to read it prior to the screening visit. Participants were encouraged to ask questions about the information provided before giving their informed consent.

During screening, participants underwent physical and mental health assessments. Physical assessments included: (i) an alcohol breathalyser test, (ii) a urine screen for drugs of abuse and pregnancy (where applicable), (iii) an electrocardiogram (ECG), (iv) routine blood tests, and (v) blood pressure, heart rate, height and weight recordings. Mental health assessments included (i) a neurological examination and (ii) a standard psychiatric interview, the Mini-International Neuropsychiatric Interview (MINI) version 5. Participants provided their full medical history and declared previous drug use. Screenings typically lasted 2.5 hours. After the retrieval of screening results, eligible participants were enrolled in the study and invited to return for subsequent visits.

Inclusion criteria

- Physically and mentally healthy
- Males & females
- 18-85 years old
- Good command of the English language
- No prior experience with psychedelic drugs

Exclusion criteria

- Current or previously diagnosed psychiatric disorder
- One or more immediate family members with a current or previously diagnosed psychotic disorder
- Medically significant condition that renders them unsuitable for the study (e.g., diabetes, severe cardiovascular disease, hepatic or renal failure etc.)
- Show MR contraindications (e.g., metal implants, pacemakers, claustrophobia etc.)
- Prior experience with a psychedelic drug
- Blood or needle phobia
- Show a positive pregnancy test at screening or during the study
- Excessive use of alcohol or other drugs (determined by study medic)

- No email access
- Inadequate grasp of English language
- Currently or very recently (within 6 months) involved in other studies
- Use of alcohol within 24 hours or other drugs within 7 days of scans and doses

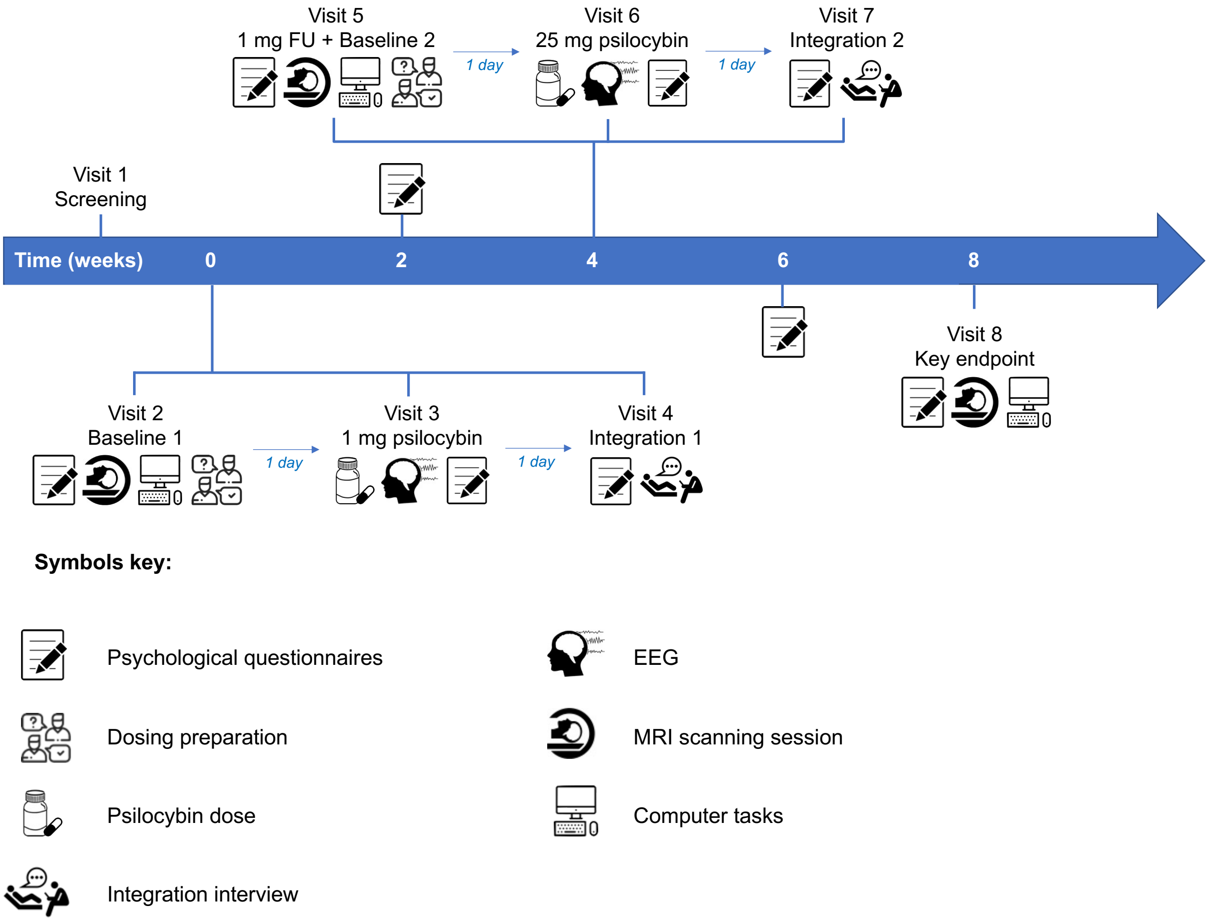

2.2. Figure S1. Study timeline of interventions. This figure shows the timeline of events in this study. Visit 2 was the true pre-intervention baseline; 1mg psilocybin dosing occurred one day later on visit 3. One-month elapsed (i.e., typically 4 weeks or just over) until visit 5, which was one day prior to 25mg psilocybin dosing (visit 6); visit 5 acted as a follow-up for the control dose of 1mg as well as a second pre-intervention (25mg psilocybin) baseline for assessing the effects of 25mg psilocybin. A further month elapsed before the key endpoint, visit 8, one-month post-25mg psilocybin. Psychological assessments were conducted on all visits as well as remotely 2 weeks after each dose. EEG recordings were completed acutely during dosing days on visits 3 and 6. Participants returned one day after each dosing day on visits 4 and 7 for a psychological integration session, involving a post-dosing check-up, open listening, and an interview about their experience. MRI scanning and a separate computer task assessing cognitive flexibility was conducted on visits 2, 5 and 8. In brief, visit 2 is baseline 1, visit 5 is baseline 2 and also the follow-up check on potential effects elicited by the control (1mg), and visit 8 is the final follow-up to assess the effects of 25 mg psilocybin.

2.3. Table S2. Schedule of interventions.

	PRE-DOSING	ACUTE	POST-DOSING		
			1 day	*2 weeks*	*1 month*
**INTENSITY**	X	X			
**EEG**	X	X			
**PIS**			X	X	X
**WEMWBS**	X			X	X
**IDED**	X				X
**MRI**	X				X

Table S2. Table showing a schedule of interventions. "Pre-dosing" refers to the pre-dosing baseline, which occurred one-day prior to the 1mg dosing session. Acute refers to measures done on the dosing day itself. Post-dosing timepoints are labeled in the Table header. Intensity refers to the subjective intensity of the drug effects verbally reported by the participants each hour after dosing, using a 0-10 scale, where '10' = the most intense effects imaginable. Abbreviations: EEG, Electroencephalography; PIS, Psychological Insight Scale; WEMWBS, Warwick-Edinburgh Mental Well-being Scale; IDED, Intra-dimensional/extra-dimensional task; MRI, Magnetic Resonance Imaging.

2.4. Table S3. Demographic data

Demographic variable	*M*	*SD (range)*
**Age** (in years)	40.6	8.7 (29-59)
	n	**%**
**Gender**		
Male	16	57%
Female	12	43%
**Ethnicity**		
Caucasian	24	86%
Undisclosed	3	11%
Black	1	3%
**Nationality**		
British	21	75%
Other	7	25%
**Education**		
Secondary School Level	12	43%
University Level	16	57%
**Employment status**		
Full time	24	86%
Part time	3	11%
Unemployed	1	3%

Table S3. Age data expressed as mean ±SD with the age range. The chi-square test was used to determine within-sample differences in categorical variables. Data expressed as frequency count (n) and percentage (%) of sample. Participants (N=28) had an average age of 41 years (*SD*=8.7, range: 29–59). Within the sample, gender (χ^2=0.57, *p*=0.450) and educational attainment (χ^2=0.62, *p*=0.430) were equally represented. All participants were naïve to psychedelic drugs and the majority were British (75%; χ^2=7.00, *p*<0.01) and Caucasian (86%; χ^2=14.57, *p*<0.001) in full-time employment (86%; χ^2=14.29, *p*<0.001).

3. EEG outcomes

3.1. Table S4. Psilocybin induces time-dependent changes in LZc and spectral power

	Doses (1mg vs 25mg) x timepoints (TPs) in hours (h) post-administration						
	**1mg x 1h**	**1mg x 2h**	**1mg x 4.5h**	**25mg x Baseline**	**25mg x 1h**	**25mg x 2h**	**25mg x 4.5h**
**LZc**	-0.01±0.01 (0.359)	-0.02±0.01 (0.036)	-0.003±0.01 (0.773)	-0.01±0.01 (0.404)	**0.05±0.01 (4x10^-4)**	**0.07±0.01 (2x10^-6)**	0.04±0.01 (0.013)
**delta**	35.8±33.5 (0.287)	25.3±33.5 (0.452)	-13.4±32.6 (0.682)	-25.4±36.1 (0.482)	-19.2±47.3 (0.685)	72.2±47.3 (0.129)	-28.7±46.4 (0.538)
**theta**	24.6±14.1 (0.082)	29.2±14.1 (0.039)	-3.5±13.7 (0.800)	-7.0±14.6 (0.631)	-47.6±19.8 (0.017)	-53.9±19.8 (0.007)	-22.9±19.5 (0.241)
**alpha**	37.9±27.1 (0.165)	67.5±27.1 (0.014)	4.7±26.4 (0.859)	4.5±29.4 (0.877)	**-163.5±38.3 (4x10^-5)**	**-223.0±28.3 (4x10^-8)**	-68.5±37.6 (0.071)
**beta**	6.51±5.14 (0.207)	6.94±5.14 (0.179)	-6.04±5.01 (0.230)	-4.42±5.33 (0.408)	-15.57±7.26 (0.034)	-7.66±7.26 (0.293)	-4.18±7.13 (0.559)
**gamma**	2.05±3.72 (0.583)	1.27±3.72 (0.732)	-6.40±3.63 (0.080)	-3.68±3.94 (0.352)	7.82±5.26 (0.140)	**18.48±5.26 (6x10^-4)**	5.59±5.17 (0.281)

Table S4. Statistics (estimates β, standard error, and uncorrected p-values, formatted β±SE (p)) of the effects of each timepoint, dose (1mg vs 25mg), and their interaction (rightmost 3 columns), as obtained by a linear mixed model. The model used timepoint and dose as independent, categorical values (with base level at baseline and 1mg, respectively), and a random intercept to account for individual differences. Each set of rows corresponds to a model with a different dependent variable, specifically LZc and spectral power in the delta, theta, alpha, beta, and gamma bands (rows along the vertical). Red font highlighted p-values are the ones that survive Bonferroni correction. D=dose (25 or 1 mg), h=hours after administration. There is a significant interaction between dose (1mg vs 25mg) and timepoint at 1hr and 2hrs on LZc and alpha. There is also a significant interaction of dose and timepoint at 2hrs for gamma. Bonferroni correction applied to 42 tests (i.e. all tests in the table, including both 6 measures and 7 terms in the LMEs.

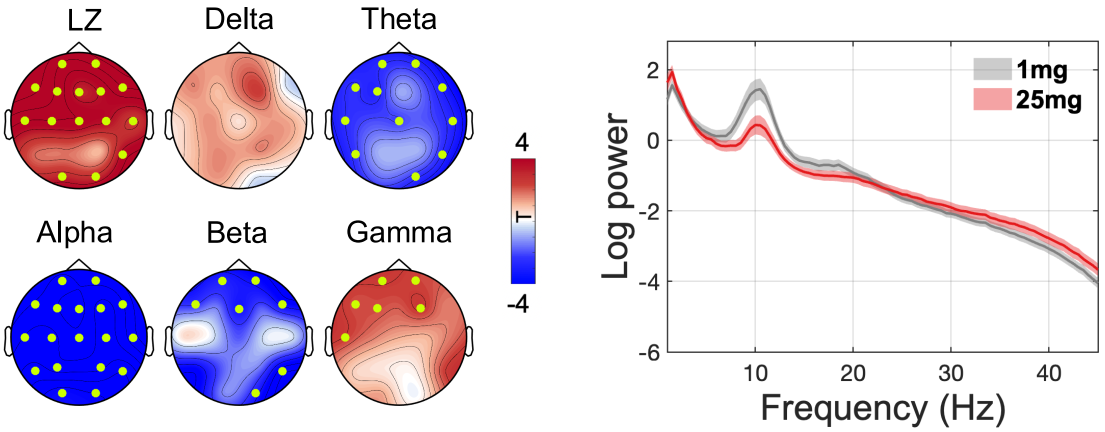

3.2. Figure S2. Sensor-level EEG maps for LZc and power in 5 canonical frequency bins. Maximum effects of 25mg vs 1 mg of psilocybin in EEG features (LZc and power in frequency bands) occurred 2-hours after administration. Localized scalp changes are marked with red electrodes are shown in the left ($p<0.05$, two-sided, cluster-corrected). Green dots indicate electrodes where there was a significant difference between the conditions. Averaged power spectra of brain activity at 2h, is shown on the right for both 25mg (red) and 1mg (blue) conditions. The spectra on the right derive from an average of sensors. Sensor-level tests corrected 19 tests.

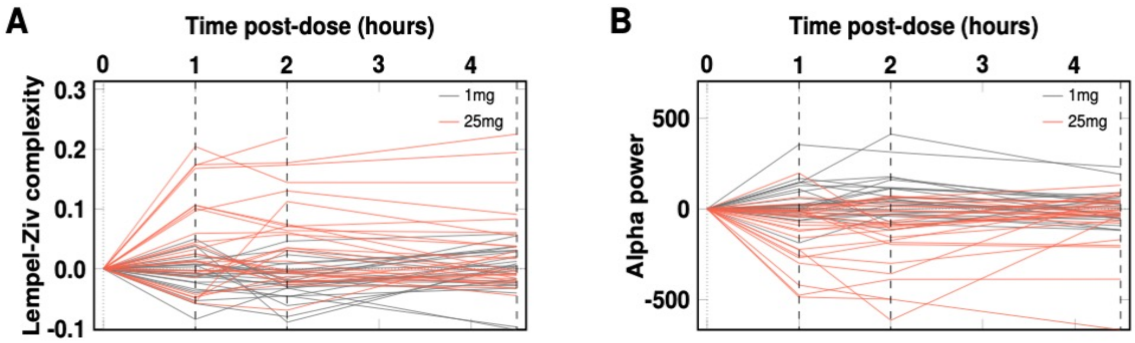

3.3. Figure S3. Single subject changes in LZc and alpha power with 1mg and 25mg psilocybin. Single subject changes in LZc (A) and alpha power (B) from pre-dose (time zero) to the 3 salient post-dose recording timepoints (1hr, 2hrs, 4.5hrs). See Figure 1 of the main manuscript to view the group level data, including variance and relevant statistical tests.

3.4 Additional controls pertaining the results of the data-driven analysis of LZc.

Here we report the findings of additional controls related to the analysis of LZc values computed from the EEG data, involving the calculation of the Spearman correlation between simple spatial averages of LZc values across electrodes obtained at different time-points and correcting for multiple comparisons via Bonferroni (see Methods). Results showed that LZc measured at 2hrs post 25mg psilocybin ingestion is significantly correlated with insight reported the day after (r=0.55, p=0.020), while LZc measured at 1hrs (r=0.41, p=0.14) and 4.5hrs (r=0.43, p=0.11) don't survive correction. These results replicate the sensor-specific results presented in the main paper (Figure 3A) wherein data from the 2hrs timepoint is shown. When running similar analyses between LZc and well-being measured one-month after, results show significant correlations between LZc measured at 1hrs (r=0.58, p=0.010) and 2hrs (r=0.54, p=0.022), but no significant correlations with LZc measured 4.5hrs (r=0.41, p=0.144). These results, obtained by much simpler means, and with a conservative multiple comparison correction, support the validity of the data-driven analyses presented in the main text. All relationships are exclusive to the 25mg dosing session.

4. fMRI outcomes

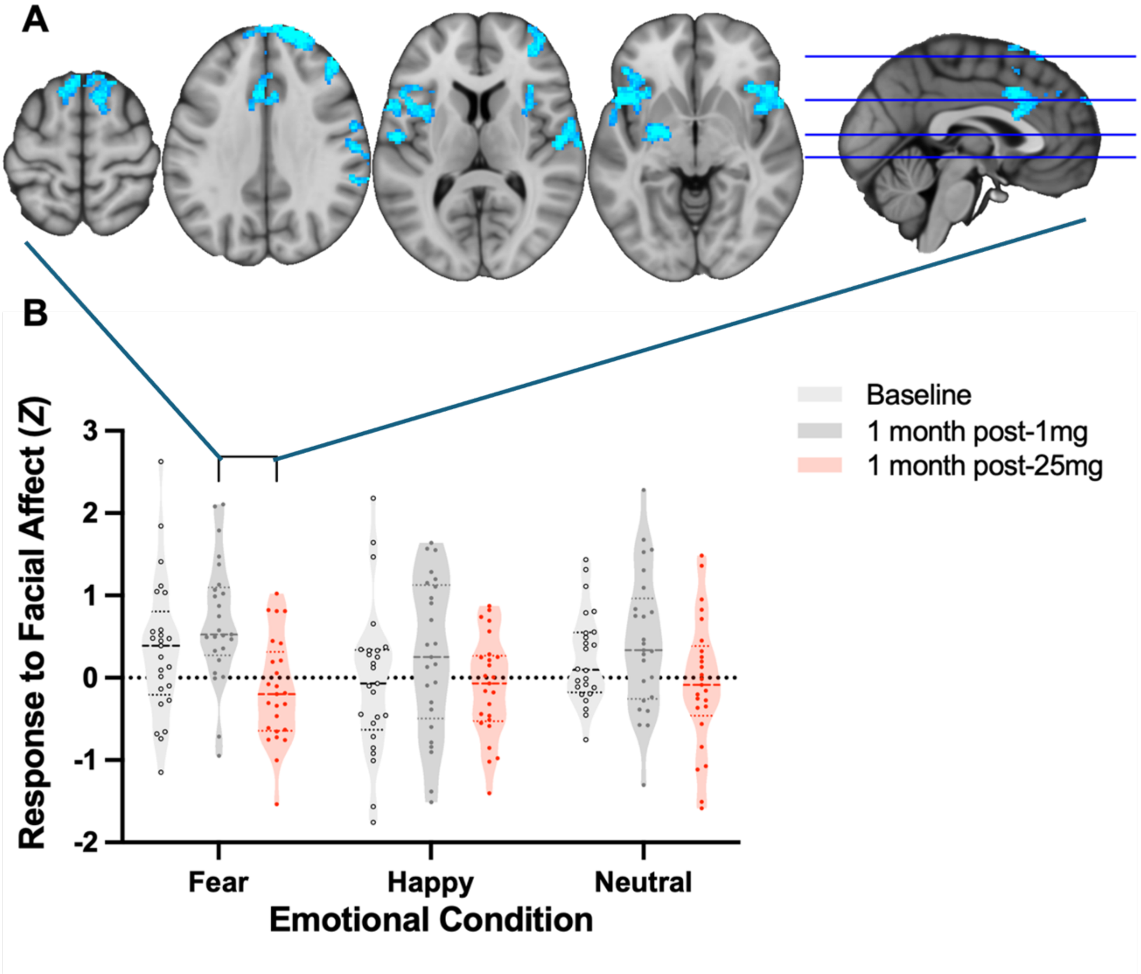

4.1. Figure S4. fMRI BOLD responses to emotional faces (all three face types).

A) Whole brain voxelwise analysis revealed significantly reduced BOLD responses to fearful faces one-month after 25mg versus one-month after 1mg ($Z = 2.3$, $p < 0.05$). This pattern of reduced responsiveness resembled the so-called 'Salience Network'. **B**) Violin plots showing BOLD responses to emotional faces of various types derived from the regions shown in Blue in A. Note, as data shown in B is derived from the one-month post-1mg vs one-month post-25mg cluster for Fear vs fixation cross (i.e., the clusters shown in A) - i.e., a test-selective cluster of voxels, we refrain from showing statistical significance tests on the values presented in this figure. Note. An inclusive ANOVA with time and emotional face type as factors failed to yield a significant result.

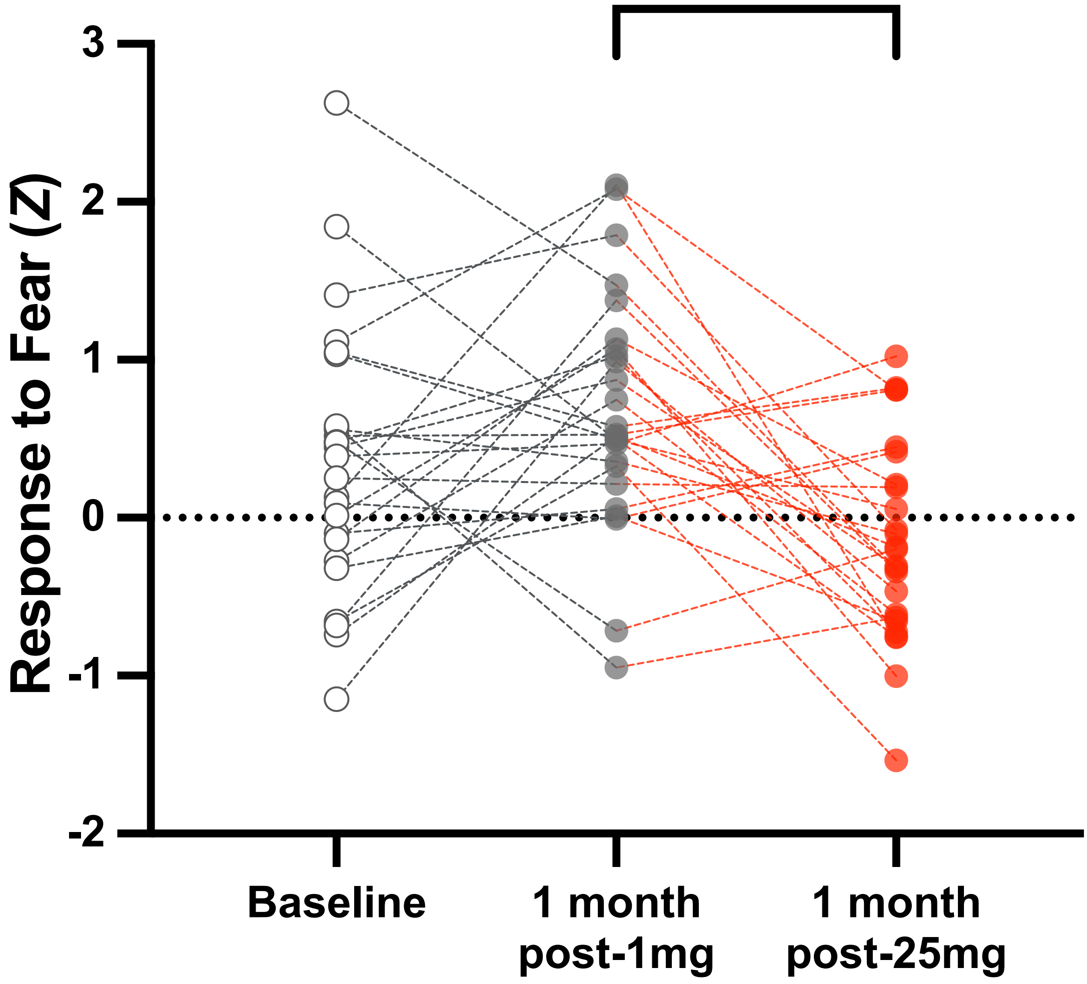

4.2. Figure S5. Response to faces with single subject values shown. Response to fear with single subject datapoints shown. These values are drawn from the cluster that was significant in the 25mg vs 1mg contrast (i.e., the cluster shown in Figure S4A - also the left-most plot of S4B). We refrain from making statistical tests on these data due to the selective derivation of the cluster and related risk of inflating the salience of an effect (that did not survive an inclusive ANOVA).

4.3. Amygdala (ROI) response to emotional faces

ROI analyses of the amygdala response (amygdala mask based on Harvard-Oxford atlas, with threshold > 50) to facial stimuli yielded a statistically significant interaction between emotional condition and time in the left amygdala ($F_{(8,192)}$=3.32, p=0.001), particularly within a distinct cluster ($F_{(8,192)}$=3.95, p<0.001); this cluster within the left amygdala is shown in Figure S4A. FDR corrected post-hoc comparisons across all timepoints and conditions showed a significant decrease in BOLD response to fearful facial stimuli one-month after 25 mg psilocybin (M_{diff}=-0.81, SE=0.30, p=0.008; 95% *CI* [0.21, 1.41], *d = 0.71).* BOLD response was also found to be significantly decreased in the Fear>Neutral+Happy (M_{diff}=-0.67, SE=0.30, p=0.029; 95% *CI* [0.07, 1.27], *d = 0.62*) and increased in the Happy>Neutral+Fear (M_{diff}=0.67, SE=0.30, p=0.028; 95% *CI* [0.07, 1.27], *d = 0.6*) conditions. No significant changes were observed one-month after the 1 mg control dose or in the right amygdala following either dose (all p>0.05).

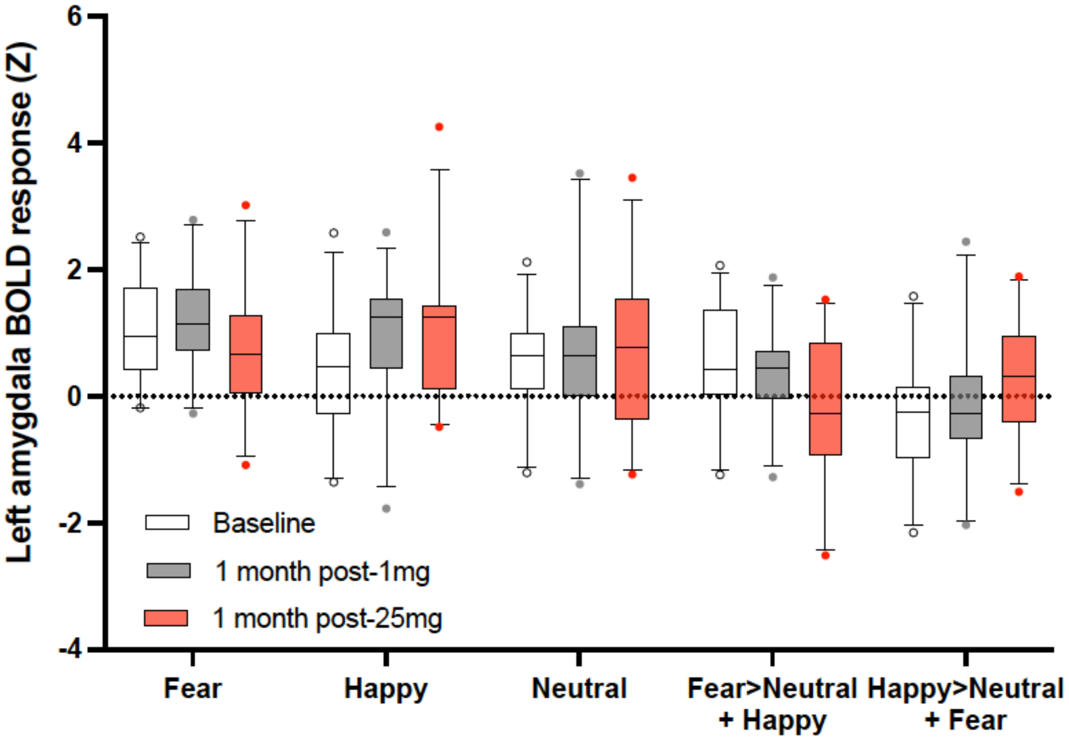

4.4. Figure S6. Box and whisker plot for the left amygdala response to emotional faces. From the Harvard-Oxford atlas, we threshold at 50% probability and then extract the values from the entire left amygdala, given that threshold. We elected not to run statistical significance tests on any paired contrasts but regardless, there was no compelling pattern of change and no effect of *Time* in an inclusive ANOVA.

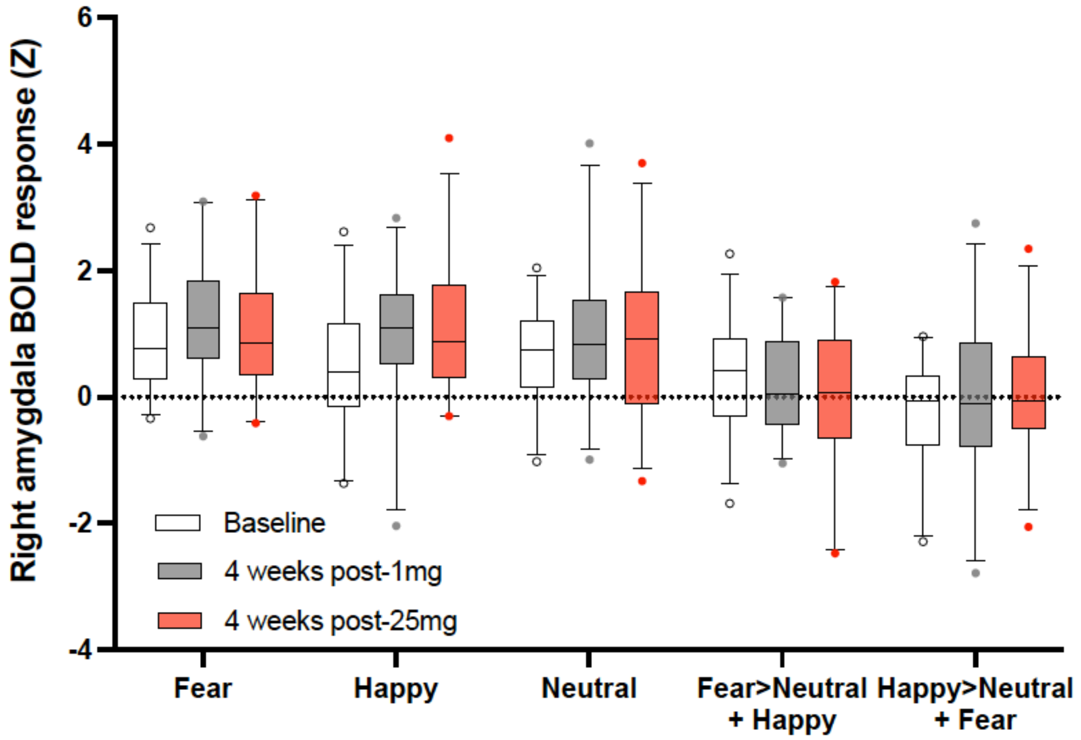

4.5. Figure S7. Box and whisker plot for the right amygdala response to emotional faces. Unlike for the left amygdala, no contrasts were statistically significant for the right amygdala. From the Harvard-Oxford atlas, we threshold at 50% probability and then extract the values from the entire right amygdala, given that threshold. As above, there were no compelling effects to report and no effect of *time* in an inclusive ANOVA.

4.6. Table S5. Effects of high-dose psilocybin on amygdala functional connectivity (general psychophysiological interaction, gPPI)

**Fear**	**Seed**	**Directionality**	**Target**	**MNI coordinates**	**Voxels**	**p-FDR cluster**
	Right amygdala	Increase	L pMTG	-50, -28, -04	151	<0.001*
		Increase	L toMTG/sLOC/AG	-54, -58, 6	71	0.019*
**Fear > Happy + Neutral**	**Seed**	**Directionality**	**Target**	**MNI coordinates**	**Voxels**	**p-FDR cluster**
	Left amygdala	Decrease	Left hippocampus	-20, -24, -8	71	0.022*
	Right amygdala	Increase	R pSMG	64, -42, 32	70	0.038
**Happy > Fear + Neutral**	**Seed**	**Directionality**	**Target**	**MNI coordinates**	**Voxels**	**p-FDR cluster**
	Left amygdala	Decrease	R occipital pole	8, -92, 16	72	0.020*
	Right amygdala	Increase	R postcentral gyrus	30, -38, 64	119	0.001*

Table S5. Effects of high-dose psilocybin on amygdala functional connectivity. *Time* contrast: post-1mg vs. post-25mg; L=left, R=right; pMTG = posterior middle temporal gyrus; toMTG = temporo-occpital MTG; sLOC = superior lateral occipital cortex; AG = angular gyrus; pSMG = posterior supramarginal gyrus; * = p-FDR<0.025 (FDR corrected at brain level and Bonferroni corrected for laterality (left and right amygdala seeds).

Psychophysiological interaction results in the face paradigm suggest emotion-specific modulation of amygdala circuitry. In the fear faces condition, amygdala functional connectivity was increased between the amygdala and temporal, parietal, and occipital areas, while amygdala-hippocampus coupling decreased. In the happy faces condition, functional connectivity was increased between the amygdala and the postcentral gyrus, while amygdala-occipital pole functional connectivity was decreased. These results are broadly consistent with a prior PPI amygdala analysis involving scanning after psilocybin-therapy for treatment-resistant depression[1], but also highlight amygdala-temporal lobe and amygdala-hippocampal functional connectivity changes that align with some previous work in depression[2,3]. However, some difference between this present study's results and prior work[1] were also apparent, such as a lack of decreased ventromedial PFC-amygdala functional connectivity.

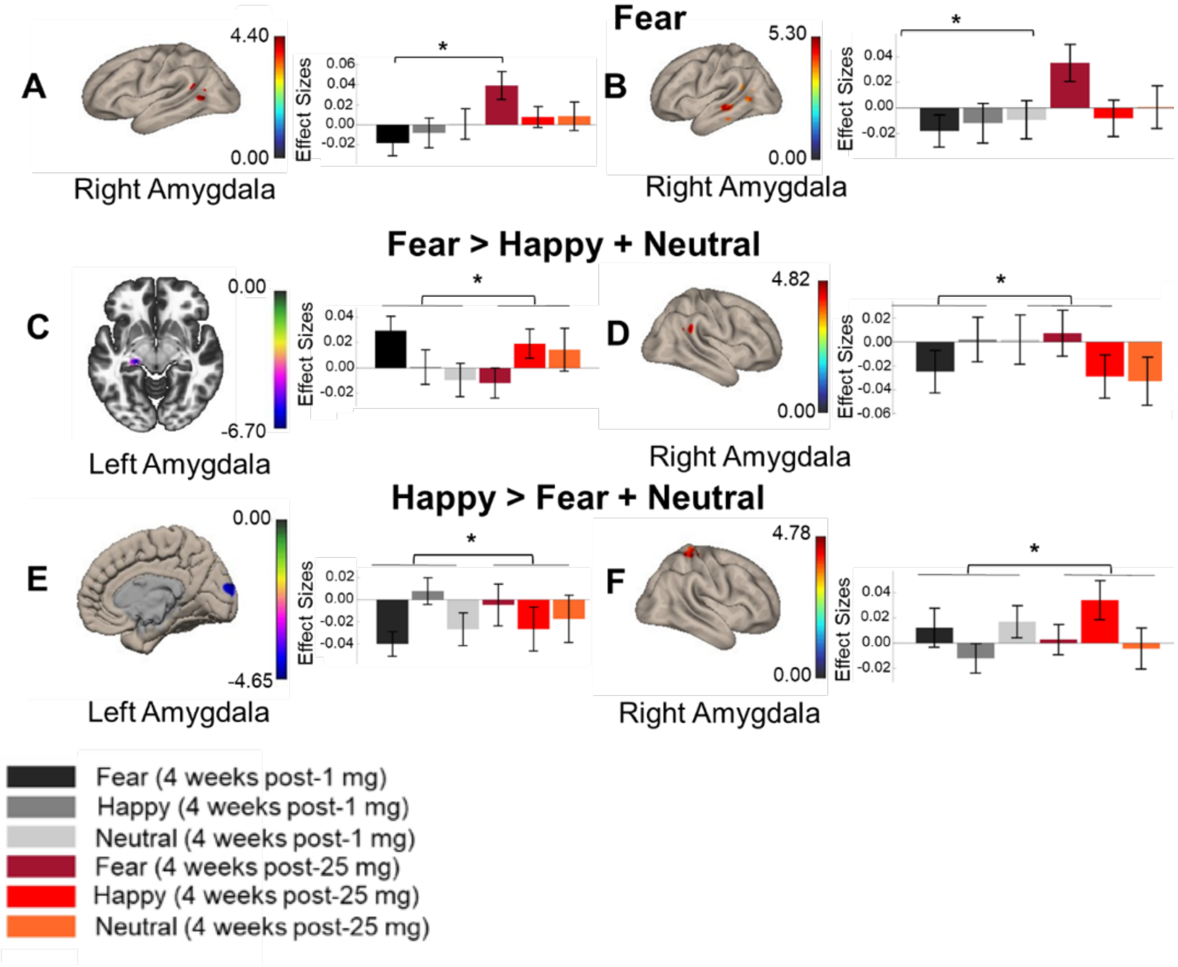

4.7. Figure S8. Effects of high-dose psilocybin on amygdala functional connectivity. A-B = fear-25mg > fear-1mg; C-D = (fear-25mg > happy-25mg + neutral-25mg) > (fear-1mg > happy-1mg + neutral-1mg); E-F = (happy-25mg > fear-25mg + neutral-25mg) > (happy-1mg > fear-1mg + neutral-1mg); A = R amygdala-posterior middle temporal gyrus, B = R amygdala-temporal occipital middle temporal gyrus, C = L amygdala-hippocampus, D = R amygdala-posterior supramarginal gyrus, E = L amygdala-occipital pole, F = R amygdala-postcentral gyrus.

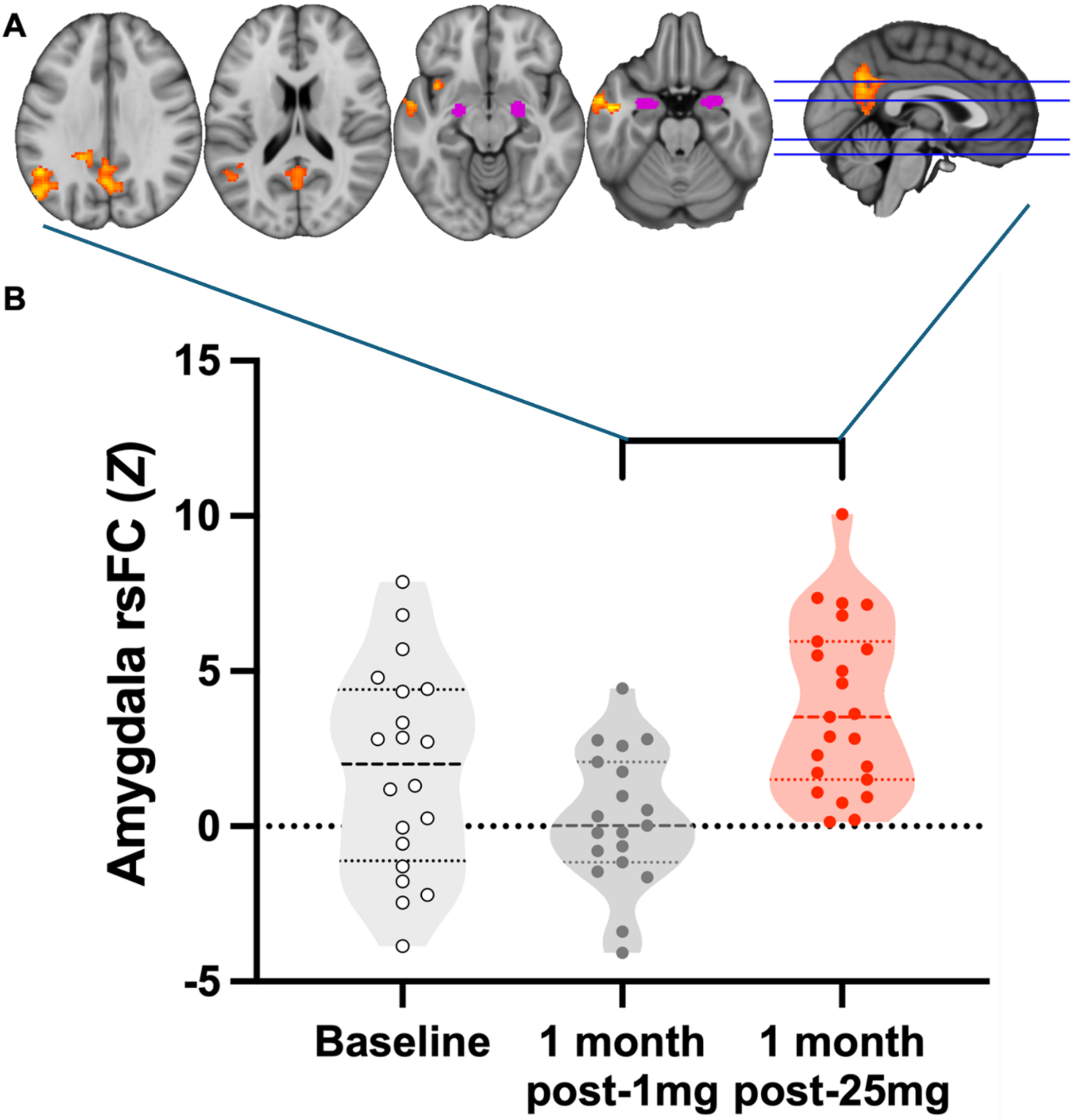

4.8. Figure S9. Bilateral amygdala BOLD RSFC analysis. A) The bilateral amygdala is shown in magenta, from the Harvard-Oxford atlas, >50% threshold. Hot colours show the cluster-corrected map (threshold $Z = 2.3$, $p < 0.05$) for amygdala RSFC contrast, one-month post-25mg vs one-month post-1mg. Increased amygdala coupling can be seen with regions that overlap the so-called 'default-mode network'. Values from this timepoint-specific contrast, and the clusters therein, contributed to the amg-RSFC correlation matrix shown in Figure 3B of the main paper. **B)** Violin plot of bilateral amygdala BOLD RSFC analysis. Coupling strength (Z stat) values are derived from a mask of the positive result for the contrast one-month post 25mg vs 1 month post 1mg, as shown in **A**. We refrain from statistical tests on these contrast-selective voxels from which the values in **B** derive. Note that this analysis is repeated as an ANOVA across all timepoints in **Figure S11, below.** That inclusive ANOVA yielded a significant effect of *time*.

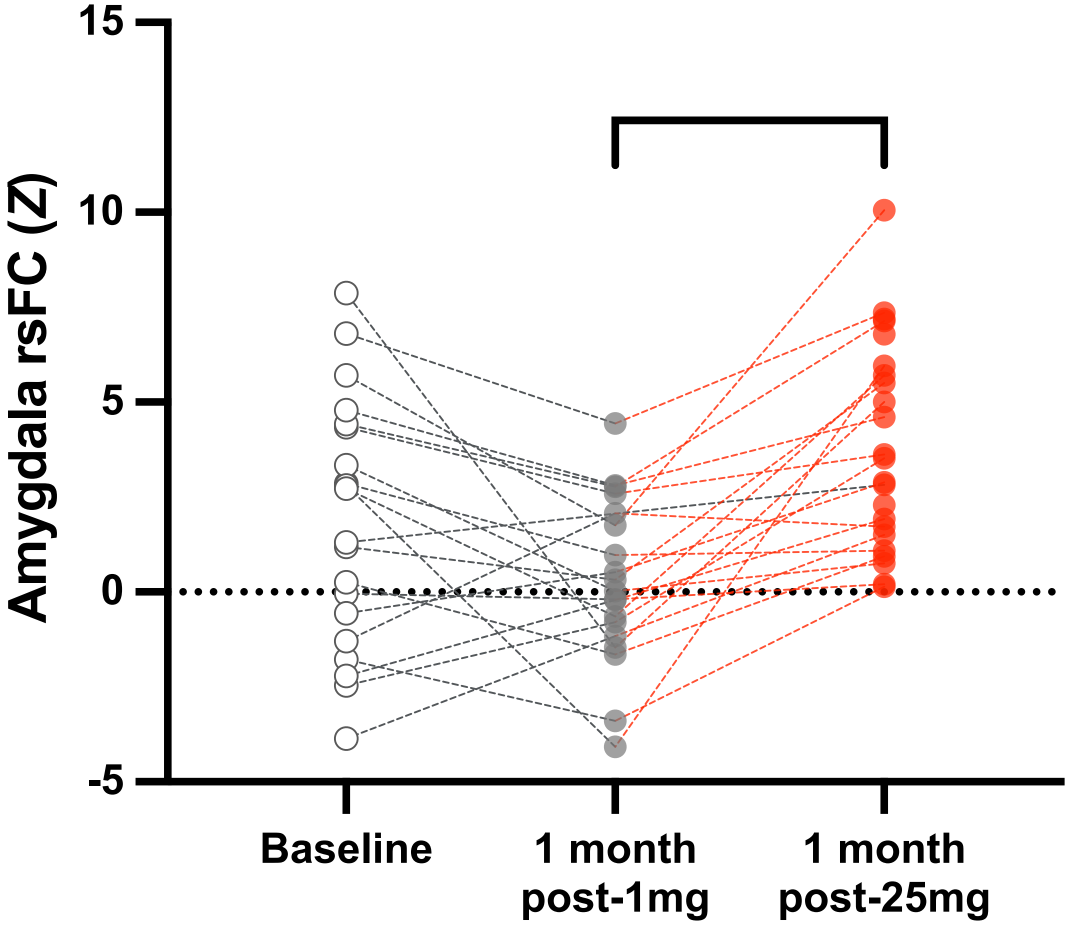

4.9. Figure S10. Amygdala RSFC with single subject values shown. This figure is intended for descriptive purposes only, to show the single subject values. Note that values derive from the cluster that was significant in the one-month post 1mg versus one-month post 25mg contrast. Note that this analysis is repeated as an ANOVA across all timepoints in Figure S11, below.

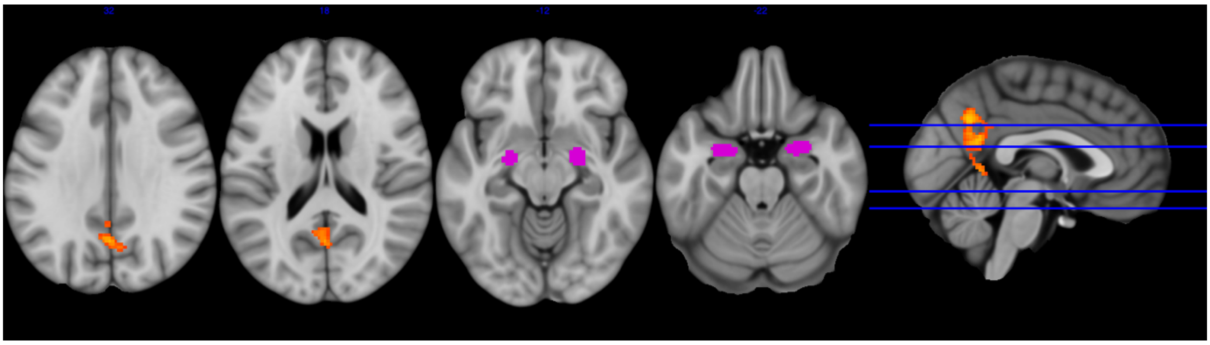

4.10. Figure S11. Amygdala seed based RSFC. ANOVA across all timepoints. The above map shows different views of the bilateral amygdala seed (magenta) in a RSFC analysis across all three timepoints, where the cluster ($z = 2.3$, whole brain, cluster corrected, $p < 0.05$) was sensitive to an interaction with *time* i.e., baseline, post-1mg, post-25mg. See Figure S10 for *time* parsed into discrete timepoints with single subject values shown.

4.11. Validation analysis: in-scanner anxiety: amygdala RSFC analysis

For the amygdala ROI rsFC analysis, we examined in-scanner visual analogue scale (VAS) ratings completed immediately after each resting state run revealed a significant effect of anxiety ($F(1.16,28.42)=7.08$, $p=0.010$), with significantly greater anxiety among the participants at pre-intervention baseline relative to one month after 1 mg psilocybin; Mdiff=1.00, SE=0.40, $p=0.013$; 95% CI [0.18, 1.82], $d=0.7$) and one month after 25mg; Mdiff=1.14, SE=0.33, $p=0.010$; 95% CI [0.33, 2.19], $d=0.7$). When anxiety levels during the baseline scan were entered into regression models as a covariate, the one-month post-1mg versus baseline scan changes in amygdala rsFC no longer reached significance ($F(1,19)=1.35$, $p=0.259$ ns) – implying that any initial observations of change in amygdala RSFC post 1mg may have been related to unusually high anxiety levels for the baseline scan (in this resting-state scan only), rather than e.g., a direct effect of 1mg psilocybin itself.

Further strengthening this inference on the role of in-scanner anxiety in the baseline scan as a confounding variable, a correlation was found between the drop in anxiety in the one-month post-1mg scan (vs the baseline scan) and parallel changes in amygdala rsFC ($R2=0.206$, $p=0.026$). Outlier analyses revealed three outliers with especially high anxiety ratings (i.e., a VAS score of >60%) for their baseline scan. When these were removed, and the analyses repeated, Bonferroni-corrected post-hoc comparisons showed a robust increase in amygdala RSFC one month after 25mg psilocybin (Mdiff=3.42, SE=0.65, $p<0.0001$; 95% CI [1.78, 5.01], $d=1.2$), and no changes one-month after the 1mg dose. We therefore decided to use this result for the main amygdala RSFC analyses shown here in this supplement (e.g., Figure S14-16); these are also the data used in the correlation plot in the main paper.

4.12. Resting State Networks (RSN) – Within and Between Networks RSFC

RSNs were derived using Independent Component Analysis (ICA) performed on Human Connectome Project data (Van Essen et al., 2013). In summary, 20 independent components (ICs) were derived, of which the same 12 functionally meaningful RSNs were identified, namely: medial visual network (VisM), lateral visual network (VisL), occipital pole network (VisO), auditory network (AUD), sensorimotor network (SMN), default-mode network (DMN), parietal cortex network (PAR), dorsal attention network (DAN), salience network (SAL), posterior opercular network (POP), left frontoparietal network (lFPN) and right frontoparietal network (rFPN). (See Roseman et al. (2014) for more details).

Network integrity (Within-RSN rsFC) was calculated for each RSN for both pre-treatment and post-treatment. All 20 HCP ICA components were entered into FSL's dual regression analysis (Beckmann et al., 2009). The first step of the dual regression used the components as regressors applied to the 4D BOLD datasets for each subject, resulting in a matrix of time-series for each ICA. The second step involved regressing these time-series into the same 4D scan data to get a subject-specific set of spatial maps (parameter estimate (PE) images). For each subject and for each condition, within each of the 12 RSNs of interest (threshold = 3), the mean PE across voxels was calculated. This mean PE represents the integrity value. Subsequently, paired t-tests were used to calculate the difference in integrity between conditions for each RSN (Bonferroni corrected for 12 RSNs).

Between-RSN rsFC was calculated in a similar manner to previous analyses involving acute LSD (Carhart-Harris et al., 2016) and psilocybin (Roseman et., 2014). Specifically, a 12 × 12 matrix was constructed representing rsFC between different RSN pairs. For each subject and for each condition, the time-series for the relevant pair of RSNs, was entered into a GLM, resulting in a PE value representing the strength of functional connectivity between them. GLM was used rather than correlation coefficients because differences between Pearson's correlations could be a result of either signal or noise differences; therefore, it is preferable to perform regression and look for differences on the PE (Friston, 2011). The GLM was estimated twice: 1) each RSN as a dependant variable in one model, and 2) each RSN as an independent variable in the second model. These two PE values were then averaged together, to generate a symmetric 12 × 12 matrix. Paired t-tests (two-tailed) were used to compare the PE values of scan 2 and scan 3. There were no significant changes for both Within-RSN RSFC, and Between-RSN RSFC.

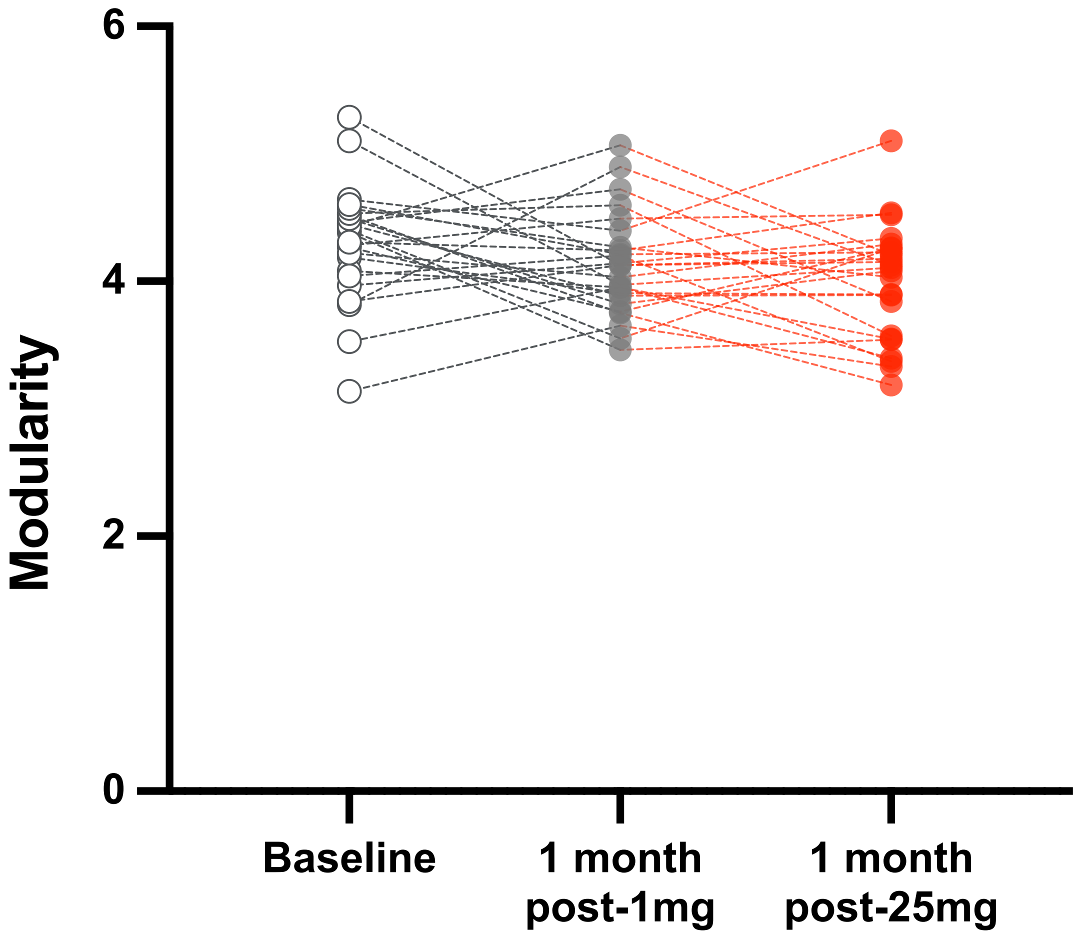

4.13. Figure S12. Modularity with single subject values shown. Modularity with single subject values shown and lines connecting all timepoints. As shown in the main manuscript, Baseline versus 1-month post-25mg yielded a significant contrast (($t(24) = -2.95$, $p = 0.007$; $d=0.6$)) but otherwise, statistical tests (ANOVA and t-tests) were negative.

4.14. An overview of fMRI results

Functional MRI findings in this study were largely negative or yielded results that while significant after appropriate correction for that specific outcome or metric, would not survive correction for all the entire range of metrics examined. More specifically, we saw reductions in amygdala and salience-network responses to fearful emotional stimuli post-25mg psilocybin in the present study (Figure S8-11) and these are broadly consistent with prior work in healthy volunteers [4]. Psychophysiological interaction results were also observed for the bilateral amygdala within the face paradigm (see S12-13). Briefly, we note some similarities and differences between a prior study examining amygdala PPI responses to emotional faces post psilocybin-therapy for depression [1]. Also note, S10&11 (happy > neutral and fear) and two prior psilocybin studies in depression [5,6] for evidence of *augmented* brain reactivity to emotional face stimuli post-psilocybin-therapy, most reliably in the non-negative emotional domains – i.e., happy and neutral faces. These latter results could be interpreted as consistent with the re-opening critical periods for social learning [7]. However, some have questioned the reliability of brain responsiveness to emotional faces as a biomarker of therapeutic action [8].

5. DTI outcomes

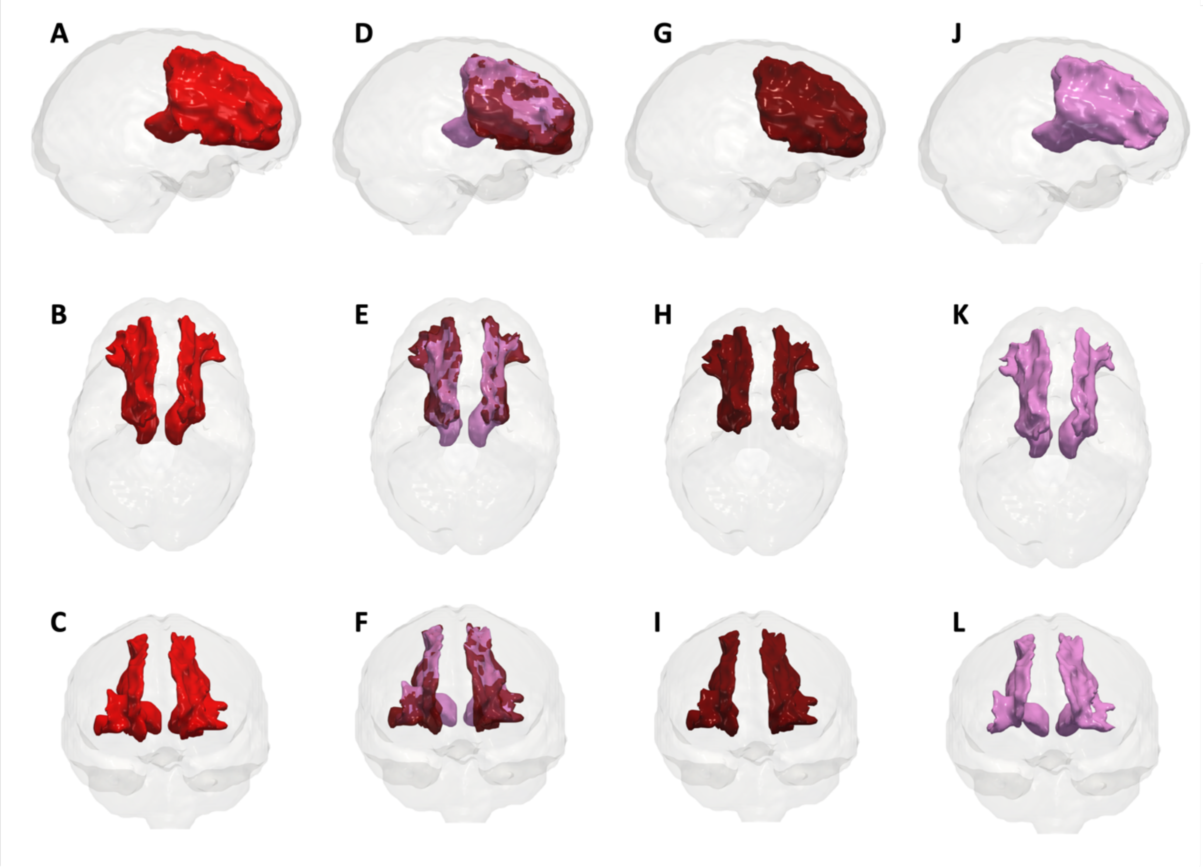

5.1. Figure S13. DTI: tracts where changes were observed (Prefrontal cortex to subcortical tracts): PFC = prefrontal cortex; THA = thalamus; STR = striatum. A-C) Combined PFC-THA & PFC-STR tracts; D-F) Combined PFC-THA (pink) & PFC-STR (dark red) tracts; G-I) PFC-STR tracts - alone; J-L) PFC-THA tracts - alone. Top-to-bottom, sagittal, axial, and coronal planes. All tracts where there was a significant decrease in axial diffusivity one-month after 25mg psilocybin.

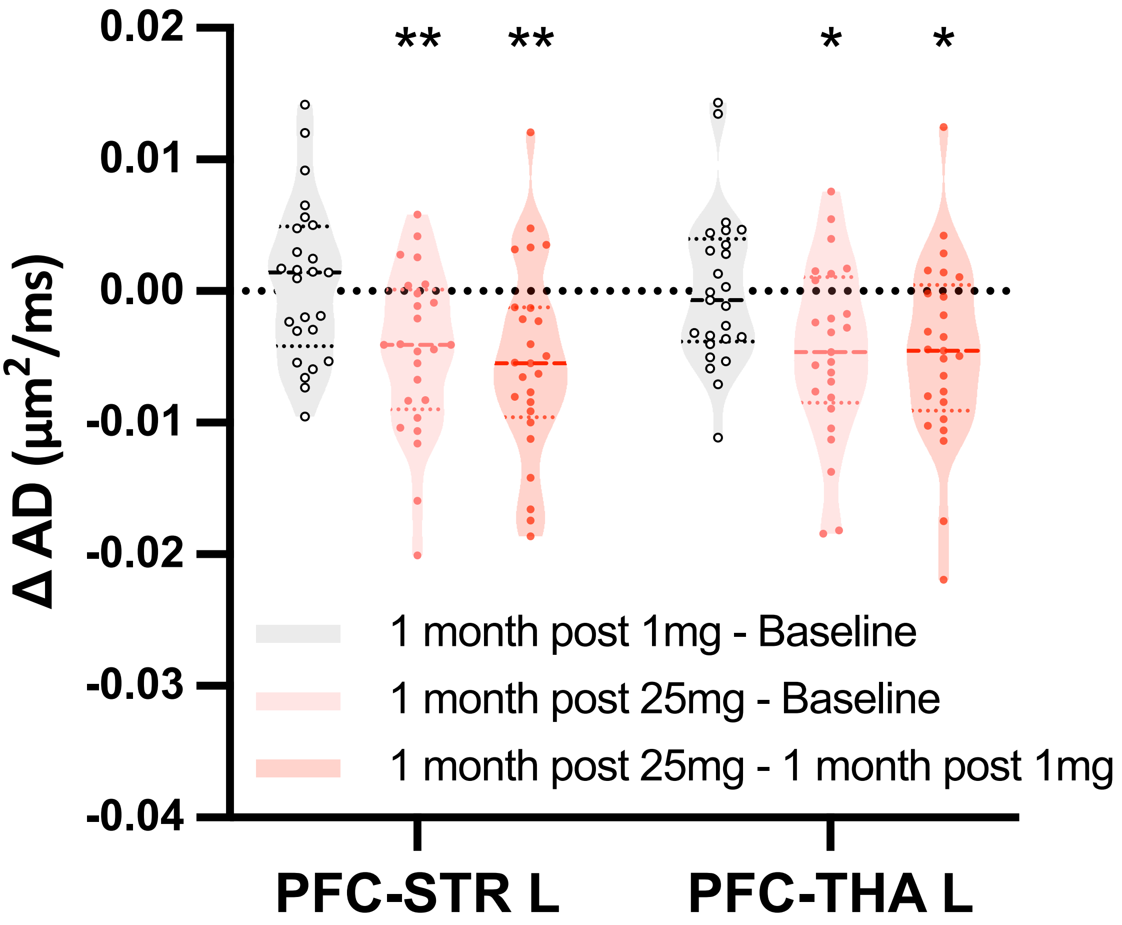

5.2 Figure S14. Axial diffusivity values without free-water correction. Left hemisphere. Post-hoc t-tests revealed a significant decrease in AD one-month post-25mg vs. one-month post-1mg in PFC-STR ($t(24) = -3.09$, $p = 0.030$) and PFC-THA ($t(24) = -3.83$, $p = 0.005$). Change in mean axial diffusivity (AD) in the left prefrontal-thamalus (PFC-THA) tract without free-water correction is shown in the violin plot above. The change in the right hemisphere was in the same direction but was not statistically significant after correction for multiple comparisons. * = $p < 0.05$, ** = $p < 0.01$, corrected for multiple comparisons.

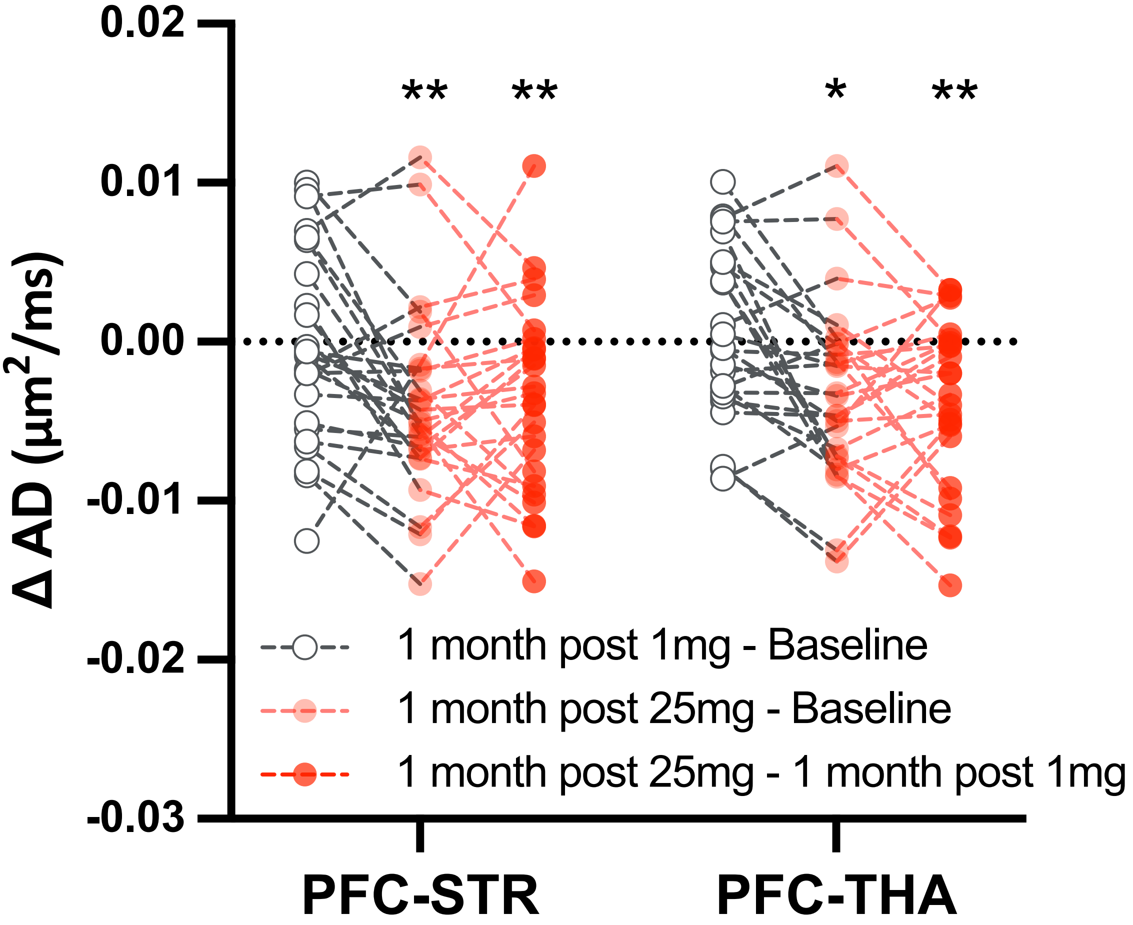

5.3. Figure S15. Axial diffusivity values with single subject datapoints shown. Axial diffusivity but with single subject datapoints shown (without free-water correction, hemispheres merged). Note that all datapoints are deltas i.e., subtractions of one timepoint from another. No changes were observed after the 1mg psilocybin (white circles). Changes only appeared after the 25mg dose of psilocybin (faint red and full red circles). * $p < 0.05$, ** $p < 0.01$.

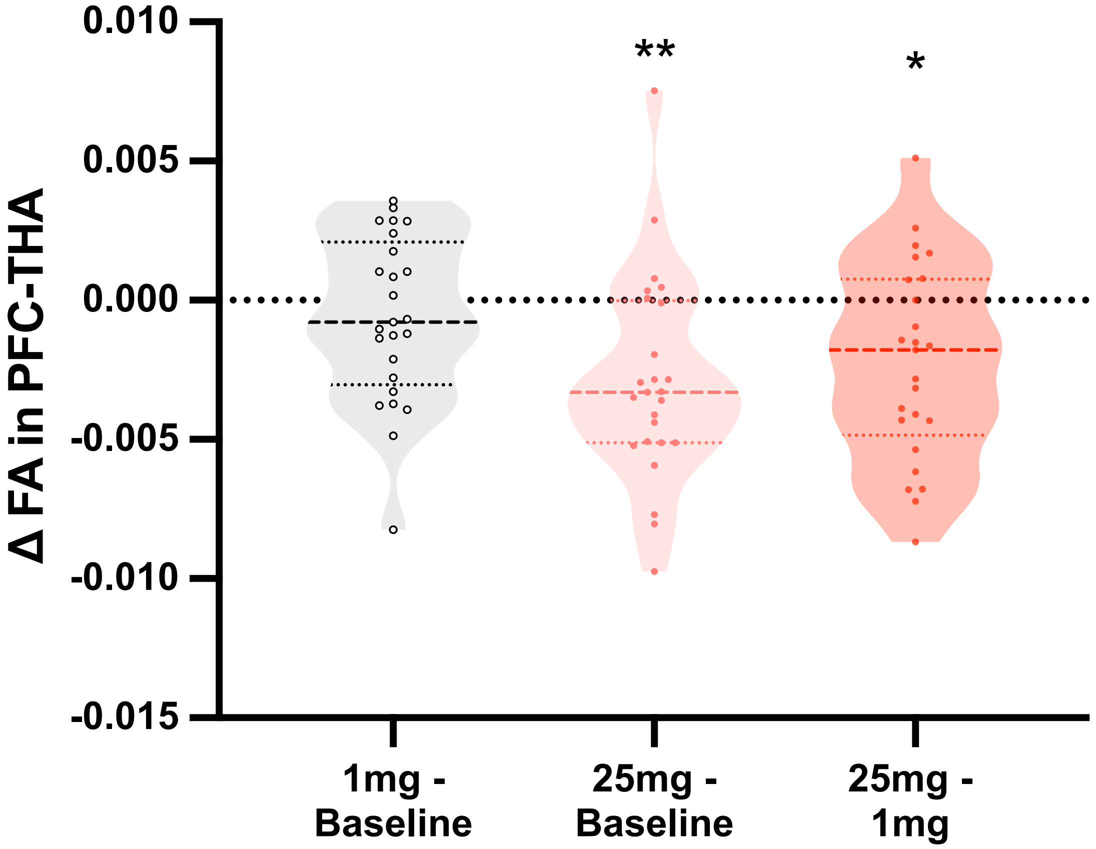

5.4. Figure S16. Fractional Anisotropy (FA) with free-water correction. Change (decreases) in mean free-water corrected fractional anisotropy (FA) in the bilateral prefrontal-thalamus (PFC-THA) tracts one-month post-25mg. Only post-25mg contrasts were significant, again implying that the DTI change was dependent on the high-dose of psilocybin. As with the other DTI metrics, there was no change after the 1mg dose (leftmost violin in grey). * = $p < 0.05$, ** = $p < 0.01$, corrected for multiple comparisons.

ANOVAs on Free-water-corrected DTI revealed a significant effect of time on FA in the PFC-STR tract ($F(2, 48) = 7.88$, $p = 0.026$) and PFC-THA tract ($F(2, 48) = 9.98$, $p = 0.006$), as well as the genu of the corpus callosum ($F(2, 48) = 7.09$, $p = 0.048$). Post-hoc t-tests with revealed a significant decrease in FA one-month post-25mg vs. one-month post-1mg in PFC-THA ($t(24) = -3.19$, $p = 0.035$).

5.5. Free-water correction values for axial diffusivity

We observed a consistent change in AD with free-water-corrected DTI. ANOVA revealed a *Time* x *Dose* interaction on AD in PFC-STR ($F(2, 48) = 10.27$, $p = 0.005$) and PFC-THA ($F(2, 48) = 12.79$, $p = 0.001$.

6. Psychological outcomes

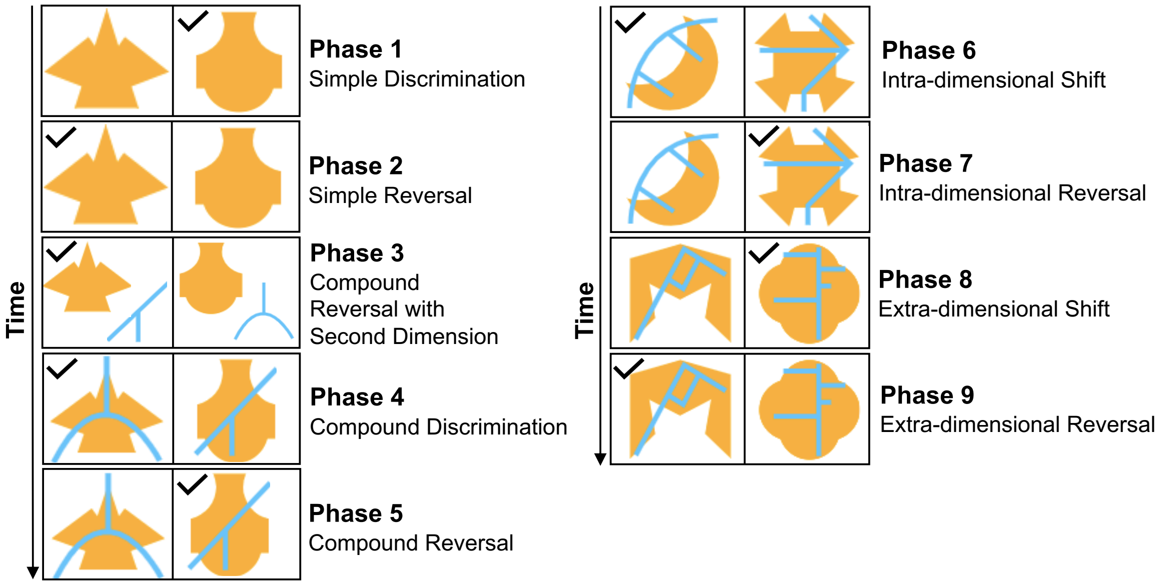

6.1. Figure S17. IDED task phase 1-9. Depiction of the nine phases of the IDED task, which assesses cognitive flexibility. The phases of the task are as follows: (1) *Simple discrimination*: Basic trial and error requiring participants to discriminate between one of two stimuli in the same dimension (yellow shapes). This phase acts to determine the initially correct stimulus; (2) *Simple reversal*: Reversal of correct stimulus, whereby previously identified stimulus will now prompt a failure notification; (3) *Compound discrimination with second dimension*: The addition of a second compound dimension, a distracting element (blue lines). The correct response remains the pre-existing stimulus (yellow shape), and therefore the new element must be ignored; (4) *Compound discrimination*: Two dimensions (yellow shapes and blue lines) become overlapped, with correct stimulus of the pair (yellow shapes) unchanged; (5) *Compound reversal*: Correct choice remains in the same dimension (yellow shapes) but moves to the other element of the pair; (6) *Intra-dimensional shift*: The illustrative stimuli change, but the correct response remains within the same dimension (yellow shape). This phase acts as a specific measure of attentional set-shifting; (7) *Intra-dimensional reversal*: Correct choice becomes the second element of the pair within the same dimension (yellow shapes); (8) *Extra-dimensional shift*: The illustrative stimuli change again, with the correct stimulus now being within the opposite dimension (blue lines). This phase examines both attentional set-shifting and reversal learning; (9) *Extra-dimensional reversal*: The correct stimulus swaps to the opposite element of the pair in the same dimension (blue lines).

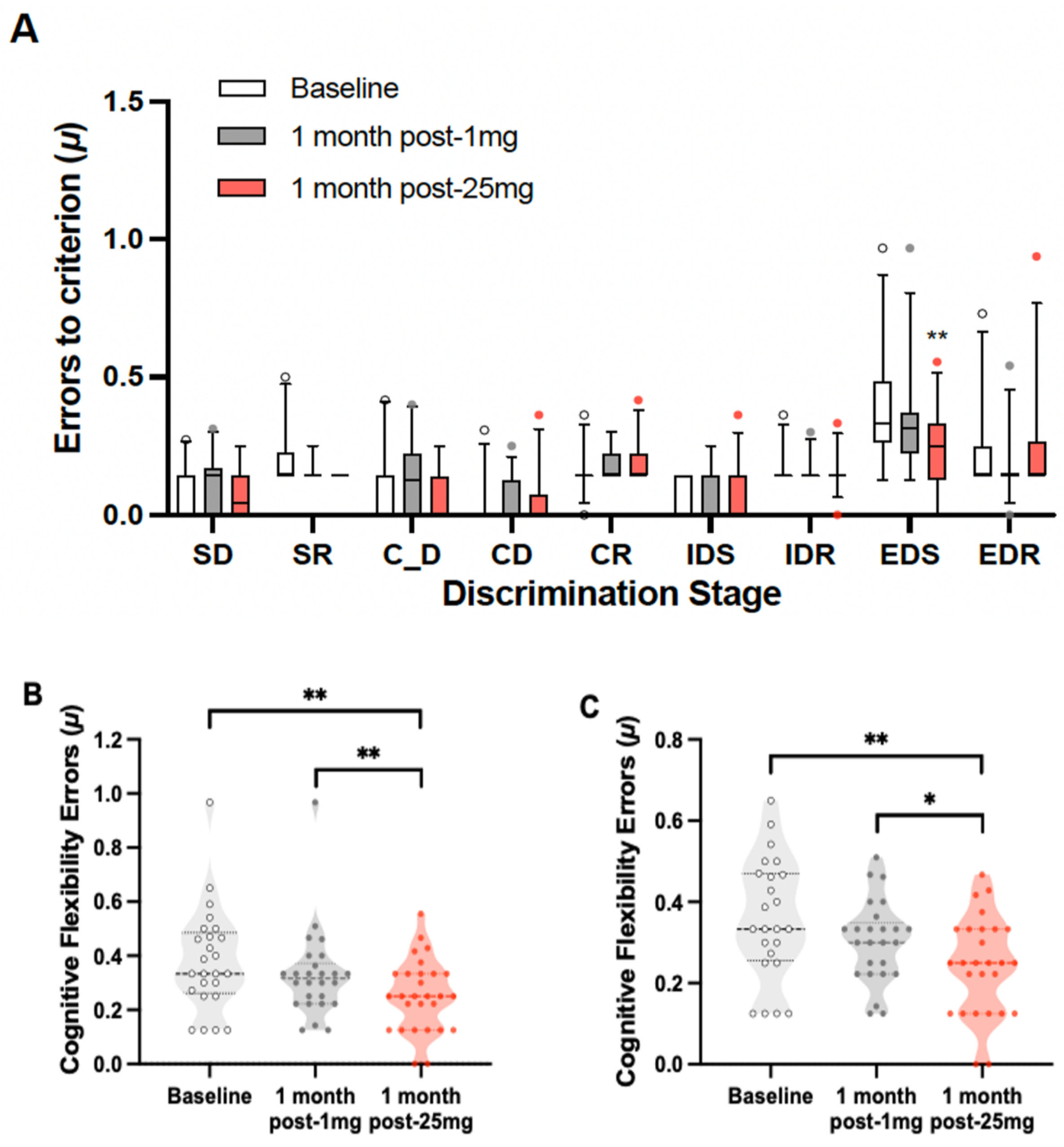

6.2. Figure S18. IDED. Cognitive flexibility (more granular) findings. A) The IDED with its various sub-parameters shown. Abbreviations: Intra-dimensional/extra-dimensional task; SD, simple discrimination; C_D, compound discrimination with second dimension; CD, compound discrimination; IDS, intra-dimensional shift; EDS, extra-dimensional shift: SR, simple reversal; CR, compound reversal, IDR, intra-dimensional reversal; EDR, extra-dimensional reversal. EDS is considered the most relevant index of cognitive flexibility; this is the parameter we measure and refer to as “cognitive flexibility errors” in B & C. ** = p < 0.01. **B**) EDS with all data included, including an outlier participant (M_{diff}=0.13, SE=0.04, p=0.008; 95% *CI* [0.02, 0.12], d=0.6). ** = p < 0.01. **C**) We also conducted an outlier analysis using the ROUT method (for ‘**R**obust regression and **Out**lier removal’) which identified one significant outlier across two timepoints (i.e., pre-dose baseline and one-month post-1mg).

Figure C therefore shows the results with this outlier removed. As this outlier scored in the direction of our findings, we report C in the main manuscript (M_{diff}=0.06, SE=0.02, p=0.016; 95% *CI* [0.00, 0.12], d=0.5), i.e., we report the more conservative analysis in the main paper and show here how the main result **was not** driven by the outlier. * = p < 0.05, ** = p < 0.01.

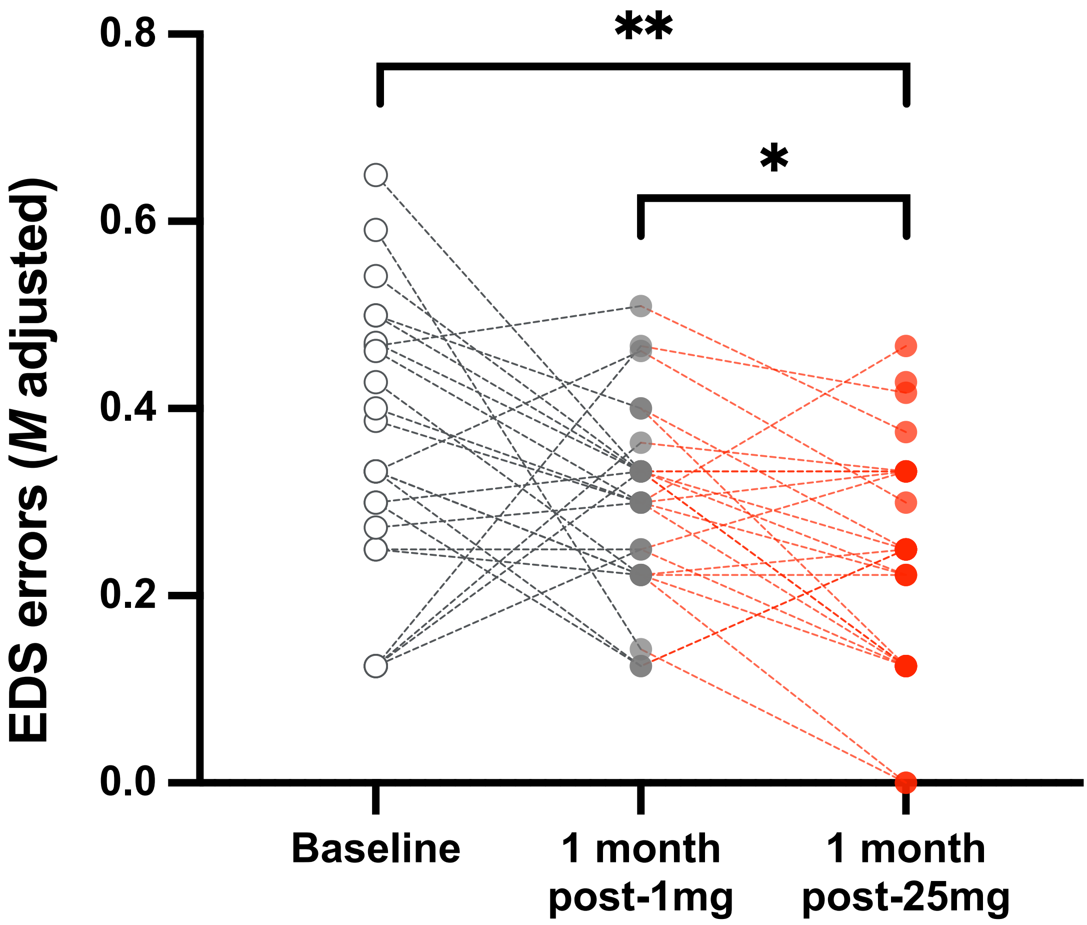

6.3. Figure S19. EDS errors. Single subject data. EDS errors showing single subject scores. Asterisks show significance at the group mean level, i.e., * p< 0.05, ** p< 0.01.

IDED task stages		Baseline	1 mg + 4 weeks	25 mg + 4 weeks
		Adjusted Errors (μ) ± μ*SEM*		
Discrimination	**SD**	0.099 ±0.027	0.111 ±0.021	0.076 ±0.016
	**C_D**	0.137 ±0.036	0.126 ±0.027	0.079 ±0.018
	**CD**	0.034 ±0.015	0.045 ±0.014	0.045 ±0.017
	**IDS**	0.068 ±0.014	0.070 ±0.016	0.076 ±0.018
	**EDS**	0.383 ±0.038	0.329 ±0.032	0.254 ± 0.025
Reversal	**SR**	0.195 ±0.018	0.155 ±0.007	0.143 ±0.000
	**CR**	0.156 ±0.012	0.172 ±0.010	0.183 ±0.013
	**IDR**	0.160 ±0.010	0.156 ±0.007	0.154 ±0.010
	**EDR**	0.217 ±0.028	0.164 ±0.018	0.248 ±0.035

6.4. Table S6. IDED task errors per criterion. Values for the IDED. All values are mean errors per criterion, adjusted for learning rate. Abbreviations: IDED, Intra-dimensional/extra-dimensional task; SD, simple discrimination; C_D, compound discrimination with second dimension; CD, compound discrimination; IDS, intra-dimensional shift; EDS, extra-dimensional shift: SR, simple reversal; CR, compound reversal, IDR, intra-dimensional reversal; EDR, extra-dimensional reversal.

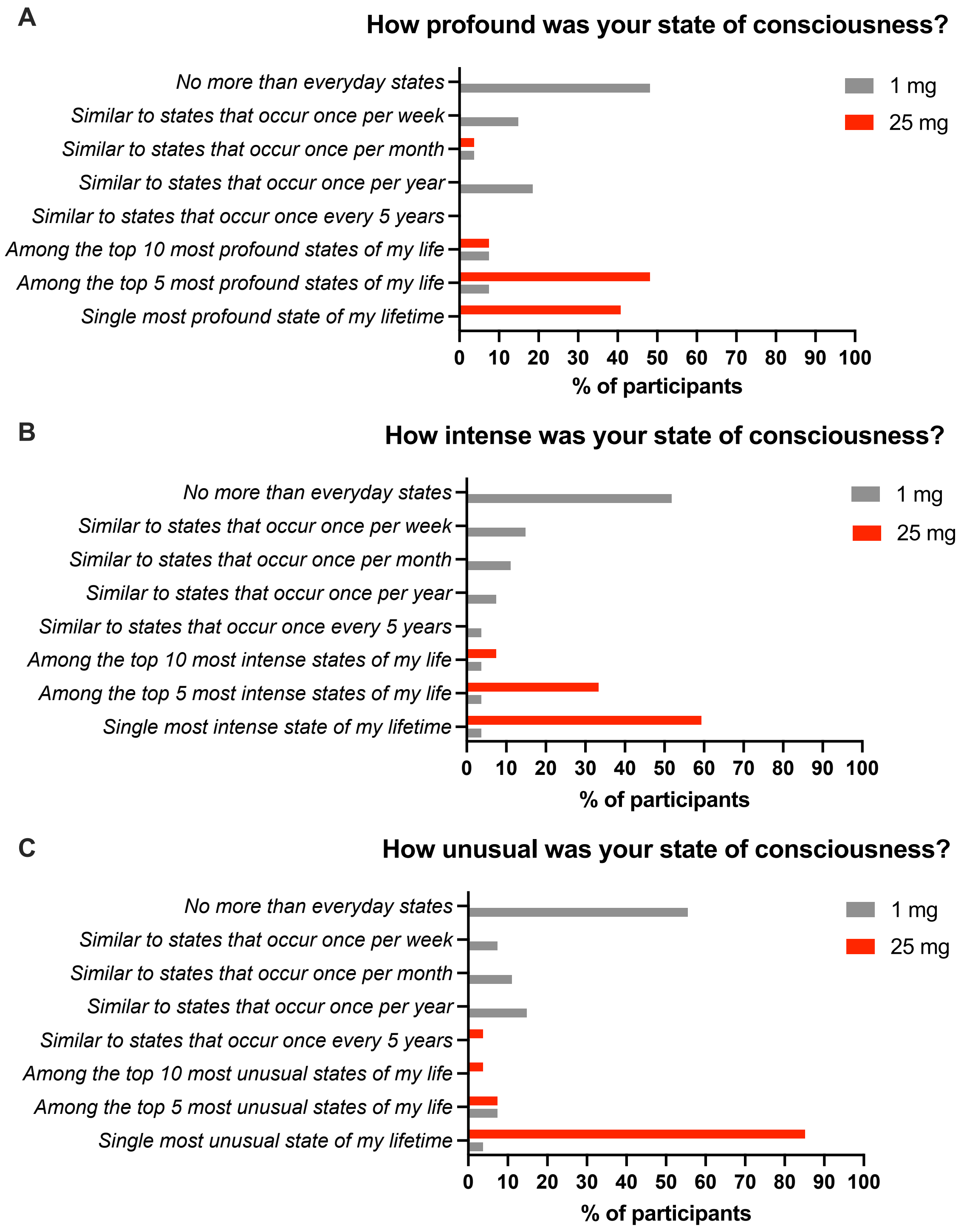

6.5. Figure S20. Adapted version of 'Persisting Effects Questionnaire' of Griffiths et al. 2006. One-month after each dosing session, we asked all participants to assess the quality of the state of consciousness they had experienced during each dosing session using the labelled ranking criteria shown on the y-axis of the above charts. Participants ranked how: **A)** *profound*, **B**) *intense*, and **C**) *unusual* their state of consciousness was in relation to their life up to that moment. They did this after both the 1mg (gray) and 25mg psilocybin experiences (red). This scale was an adapted version of the Persisting Effects Questionnaire used in [9].

7. Sample Size

7.1 Table S7. Sample size included in analyses per metric.

**Metric**	***n***	***Reason for exclusions***
Lempel-Ziv Complexity (LZc)	22	n=6 excluded due to < 60% clean trials.
Spectral Power (EEG)	22	n=6 excluded due to < 60% clean trials.
Diffusion Tensor Imagine (DTI)	25	n=3 incomplete scans due to Covid lockdown
fMRI response to emotional face stimuli	25	n=3 incomplete scans due to Covid lockdown
Resting-state Functional Connectivity (RSFC)	25	n=3 incomplete scans due to Covid lockdown
Acute subjective intensity ratings	28	N/A
Psychological insight	28	N/A
Psychological well-being	28	N/A
Cognitive flexibility	26	n=2 failed/timed-out the task

Table S7. Table showing the sample size included in each analysis and the reasons for participant exclusion.

8. References

1 Mertens, L. J. *et al.* Therapeutic mechanisms of psilocybin: Changes in amygdala and prefrontal functional connectivity during emotional processing after psilocybin for treatment-resistant depression. *J Psychopharmacol* **34**, 167-180 (2020). https://doi.org/10.1177/0269881119895520

2 Wackerhagen, C. *et al.* Amygdala functional connectivity in major depression - disentangling markers of pathology, risk and resilience. *Psychol Med* **50**, 2740-2750 (2020). https://doi.org/10.1017/S0033291719002885

3 Tang, S. *et al.* Abnormal amygdala resting-state functional connectivity in adults and adolescents with major depressive disorder: A comparative meta-analysis. *EBioMedicine* **36**, 436-445 (2018). https://doi.org/10.1016/j.ebiom.2018.09.010

4 Barrett, F. S., Doss, M. K., Sepeda, N. D., Pekar, J. J. & Griffiths, R. R. Emotions and brain function are altered up to one month after a single high dose of psilocybin. *Sci Rep* **10**, 2214 (2020). https://doi.org/10.1038/s41598-020-59282-y

5 Roseman, L., Demetriou, L., Wall, M. B., Nutt, D. J. & Carhart-Harris, R. L. Increased amygdala responses to emotional faces after psilocybin for treatment-resistant depression. *Neuropharmacology* **142**, 263-269 (2018). https://doi.org/10.1016/j.neuropharm.2017.12.041

6 Wall, M. B. *et al.* Reduced brain responsiveness to emotional stimuli with escitalopram but not psilocybin therapy for depression. *medRxiv*, 2023.2005.2029.23290667 (2023). https://doi.org/10.1101/2023.05.29.23290667

7 Nardou, R. *et al.* Psychedelics reopen the social reward learning critical period. *Nature* **618**, 790-798 (2023). https://doi.org/10.1038/s41586-023-06204-3

8 Nord, C. L., Gray, A., Charpentier, C. J., Robinson, O. J. & Roiser, J. P. Unreliability of putative fMRI biomarkers during emotional face processing. *Neuroimage* **156**, 119-127 (2017). https://doi.org/10.1016/j.neuroimage.2017.05.024

9 Griffiths, R. R., Richards, W. A., McCann, U. & Jesse, R. Psilocybin can occasion mystical-type experiences having substantial and sustained personal meaning and spiritual significance. *Psychopharmacology* **187**, 268-283; discussion 284-292 (2006). https://doi.org/10.1007/s00213-006-0457-5
