# Supplementary material for: Human brain changes after first psilocybin use
Source: Nat Commun. 2026 May 5;17:3977. doi: 10.1038/s41467-026-71962-3 (PMC13144397; doi:10.1038/s41467-026-71962-3)
Supplement: Supplementary file 2 — Reporting Summary [file 41467_2026_71962_MOESM2_ESM.pdf]

nature portfolio

Corresponding author(s): Richard Zeifman

Last updated by author(s): Mar 25, 2026

Reporting Summary

Nature Portfolio wishes to improve the reproducibility of the work that we publish. This form provides structure for consistency and transparency in reporting. For further information on Nature Portfolio policies, see our Editorial Policies and the Editorial Policy Checklist.

Statistics

For all statistical analyses, confirm that the following items are present in the figure legend, table legend, main text, or Methods section.

n/a	Confirmed	
☐	☒	The exact sample size (*n*) for each experimental group/condition, given as a discrete number and unit of measurement
☐	☒	A statement on whether measurements were taken from distinct samples or whether the same sample was measured repeatedly
☐	☒	The statistical test(s) used AND whether they are one- or two-sided<br>*Only common tests should be described solely by name; describe more complex techniques in the Methods section.*
☐	☒	A description of all covariates tested
☐	☒	A description of any assumptions or corrections, such as tests of normality and adjustment for multiple comparisons
☐	☒	A full description of the statistical parameters including central tendency (e.g. means) or other basic estimates (e.g. regression coefficient) AND variation (e.g. standard deviation) or associated estimates of uncertainty (e.g. confidence intervals)
☐	☒	For null hypothesis testing, the test statistic (e.g. *F*, *t*, *r*) with confidence intervals, effect sizes, degrees of freedom and *P* value noted<br>*Give P values as exact values whenever suitable.*
☒	☐	For Bayesian analysis, information on the choice of priors and Markov chain Monte Carlo settings
☐	☒	For hierarchical and complex designs, identification of the appropriate level for tests and full reporting of outcomes
☐	☒	Estimates of effect sizes (e.g. Cohen's *d*, Pearson's *r*), indicating how they were calculated

Our web collection on statistics for biologists contains articles on many of the points above.

Software and code

Policy information about availability of computer code

Data collection	Remote psychological assessments were collected using 'Alchemer Survey' (https://www.alchemer.com).<br>EEG: DSI-24 System, Wearable Sensing<br>MRI: 3T Siemens Tim Trio using a 12-channel head coil.
Data analysis	Statistical analyses:<br>SPSS version 26.0 (IBM Corp., Armonk, NY, USA), R Studio 2022.07.1 (RStudio PBC, Boston,. MA, USA), SciPy 1.9.1 and statsmodels 0.13.2 in Python 3.9.13 (Python Software Foundation), or MATLAB 2019b (MathWorks, Natick, USA) and illustrated using GraphPad Prism version 9.2 (GraphPad Software, La Jolla California USA).<br><br>fMRI:<br>FMRIB Software Library (FSL), Analysis of Functional NeuroImages (AFNI), Freesurfer and Advanced Normalization Tools (ANTS). The RSFC analyses were performed using FSL's FEAT for each subject. MRIcron was used to display the results.<br><br>Modularity was computed using the Louvain algorithm implemented with the Network Community Toolbox (http://commdetect.weebly.com/), treating negative values asymmetrically.<br><br>dMRI:<br>Images were preprocessed using MRtrix3. TractSeg's pre-trained neural network was used to segment white matter and its outputs, representing probabilities of the tract being present in a voxel, were converted into binary masks with a threshold of 0.975 for reliable and reproducible segmentation.

EEG:
All EEG data were preprocessed using the Fieldtrip toolbox in MATLAB (R2019B, MathWorks, Inc)

For manuscripts utilizing custom algorithms or software that are central to the research but not yet described in published literature, software must be made available to editors and reviewers. We strongly encourage code deposition in a community repository (e.g. GitHub). See the Nature Portfolio guidelines for submitting code & software for further information.

Data

Policy information about availability of data

All manuscripts must include a data availability statement. This statement should provide the following information, where applicable:

- Accession codes, unique identifiers, or web links for publicly available datasets
- A description of any restrictions on data availability
- For clinical datasets or third party data, please ensure that the statement adheres to our policy

Information required to reanalyze the data reported in this paper is available from the lead contacts upon request.

Research involving human participants, their data, or biological material

Policy information about studies with human participants or human data. See also policy information about sex, gender (identity/presentation), and sexual orientation and race, ethnicity and racism.

Reporting on sex and gender	Participants provided their gender identification, which remained the same as their sex at birth. The sample was 43% female and 57% male.
Reporting on race, ethnicity, or other socially relevant groupings	Age, ethnicity, nationality, education level and employment status are displayed in the demographics table provided in supplementary materials.
Population characteristics	See above
Recruitment	N=28 were recruited via an online advertisement on the Centre for Psychedelic Research website. All participants initiated contact to gain their place on the study. All participants were completely naive to psychedelic drugs.
Ethics oversight	This study was approved by the London-Surrey Research Ethics Committee and sponsored by the Joint Research and Compliance Office, Imperial College London. The National Institute for Health Research/Wellcome Trust Imperial Clinical Research Facility (ICRF) provided site-specific approvals.

Note that full information on the approval of the study protocol must also be provided in the manuscript.

Field-specific reporting

Please select the one below that is the best fit for your research. If you are not sure, read the appropriate sections before making your selection.

☐ Life sciences ☒ Behavioural & social sciences ☐ Ecological, evolutionary & environmental sciences

For a reference copy of the document with all sections, see nature.com/documents/nr-reporting-summary-flat.pdf

Life sciences study design

All studies must disclose on these points even when the disclosure is negative.

Sample size	*Describe how sample size was determined, detailing any statistical methods used to predetermine sample size OR if no sample-size calculation was performed, describe how sample sizes were chosen and provide a rationale for why these sample sizes are sufficient.*
Data exclusions	*Describe any data exclusions. If no data were excluded from the analyses, state so OR if data were excluded, describe the exclusions and the rationale behind them, indicating whether exclusion criteria were pre-established.*
Replication	*Describe the measures taken to verify the reproducibility of the experimental findings. If all attempts at replication were successful, confirm this OR if there are any findings that were not replicated or cannot be reproduced, note this and describe why.*
Randomization	*Describe how samples/organisms/participants were allocated into experimental groups. If allocation was not random, describe how covariates were controlled OR if this is not relevant to your study, explain why.*
Blinding	*Describe whether the investigators were blinded to group allocation during data collection and/or analysis. If blinding was not possible, describe why OR explain why blinding was not relevant to your study.*

Behavioural & social sciences study design

All studies must disclose on these points even when the disclosure is negative.

Study description	This was a multi-phase, multi-modal human neuroimaging study with the serotonin receptor agonist and experimental medicine, psilocybin. This controlled, fixed-order, within-subjects study investigated the effects of psilocybin in healthy human adults with no prior psychedelic experience (N=28). All participants received two oral doses of psilocybin, 4-weeks apart: (1) a control dose of 1mg psilocybin on the first dosing day, considered to be a subthreshold dose that is unable to occasion a psychedelic experience; and (2) a fully active dose of 25mg psilocybin, considered to be a high dose and capable of inducing profound psychedelic effects, 4-weeks or 1-month later. This fixed order design was necessary given the hypothesized carry-over effects of 25mg psilocybin. To uphold blinding and control for expectancy effects, participants were informed that they would receive psilocybin on both sessions of a variable dose up to 25mg. No further information regarding dosage was provided.
Research sample	Age (in years): M=40.6, SD= 8.7 (range = 29-59 years old)<br>Sex<br>Male: N=16<br>Female: N=12<br>Ethnicity<br>Caucasian: 24<br>Undisclosed: 3<br>Black: 1<br>Nationality<br>British: 21<br>Other: 7<br>Education<br>Secondary School Level: 12<br>University Level: 16<br>Employment status<br>Full time: 24<br>Part time: 3<br>Unemployed: 1<br><br>The chi-square test was used to determine within-sample differences in categorical variables. Data expressed as frequency count (n) and percentage (%) of sample. Participants (N=28) had an average age of 41 years (SD=8.7, range: 29–59) and were balanced in terms of gender ($\chi2=0.57$, $p=0.450$) and educational attainment ($\chi2=0.62$, $p=0.430$). All participants were naïve to psychedelic drugs and the majority were British (75%; $\chi2=7.00$, $p<0.01$) and Caucasian (86%; $\chi2=14.57$, $p<0.001$) in full-time employment (86%; $\chi2=14.29$, $p<0.001$).
Sampling strategy	Sample size exceeded that required for 80% power as determined via power analyses.
Data collection	Computer task (IDED) completed with the researcher in the room, but without oversight or interaction. Questionnaires completed without the presence of research team, with data anonymised (remotely using online platform, Alchemer Survey), EEG recordings on dosing days and MRI at baseline and 1 month follow-ups.
Timing	Visit 1 was the screening visit. Visit 2 was the true pre-intervention baseline; 1mg psilocybin dosing occurred one day later on visit 3. One-month elapsed (i.e., typically 4 weeks or just over) until visit 5, which was one day prior to 25mg psilocybin dosing (visit 6); visit 5 acted as a follow-up for the control dose of 1mg as well as a second pre-intervention (25mg psilocybin) baseline for assessing the effects of 25mg psilocybin. A further month elapsed before the key endpoint, visit 8, one-month post-25mg psilocybin. Psychological assessments were conducted on all visits as well as remotely 2 weeks after each dose. EEG recordings were completed acutely during dosing days on visits 3 and 6. Participants returned one day after each dosing day on visits 4 and 7 for a psychological integration session, involving a post-dosing check-up, open listening, and an interview about their experience. MRI scanning and a separate computer task assessing cognitive flexibility was conducted on visits 2, 5 and 8. In brief, visit 2 is baseline 1, visit 5 is baseline 2 and also the follow-up check on potential effects elicited by the control (1mg), and visit 8 is the final follow-up to assess the effects of 25 mg psilocybin.
Data exclusions	See supplementary materials (Table S7) for details surrounding sample size included in analyses per metric.
Non-participation	NA
Randomization	Controlled within-subjects study with a fixed-order design given hypothesised carry-over effects of psilocybin. All participants received two oral doses of psilocybin, 4-weeks apart: (1) a control dose of 1mg psilocybin on the first dosing day, considered to be a subthreshold dose that is unable to occasion a psychedelic experience; and (2) a fully active dose of 25mg psilocybin, considered to be a high dose and capable of inducing profound psychedelic effects, 4-weeks or 1-month later.

Ecological, evolutionary & environmental sciences study design

All studies must disclose on these points even when the disclosure is negative.

Study description	*Briefly describe the study. For quantitative data include treatment factors and interactions, design structure (e.g. factorial, nested,*

hierarchical), nature and number of experimental units and replicates.

Research sample	Describe the research sample (e.g. a group of tagged Passer domesticus, all Stenocereus thurberi within Organ Pipe Cactus National Monument), and provide a rationale for the sample choice. When relevant, describe the organism taxa, source, sex, age range and any manipulations. State what population the sample is meant to represent when applicable. For studies involving existing datasets, describe the data and its source.
Sampling strategy	Note the sampling procedure. Describe the statistical methods that were used to predetermine sample size OR if no sample-size calculation was performed, describe how sample sizes were chosen and provide a rationale for why these sample sizes are sufficient.
Data collection	Describe the data collection procedure, including who recorded the data and how.
Timing and spatial scale	Indicate the start and stop dates of data collection, noting the frequency and periodicity of sampling and providing a rationale for these choices. If there is a gap between collection periods, state the dates for each sample cohort. Specify the spatial scale from which the data are taken
Data exclusions	If no data were excluded from the analyses, state so OR if data were excluded, describe the exclusions and the rationale behind them, indicating whether exclusion criteria were pre-established.
Reproducibility	Describe the measures taken to verify the reproducibility of experimental findings. For each experiment, note whether any attempts to repeat the experiment failed OR state that all attempts to repeat the experiment were successful.
Randomization	Describe how samples/organisms/participants were allocated into groups. If allocation was not random, describe how covariates were controlled. If this is not relevant to your study, explain why.
Blinding	Describe the extent of blinding used during data acquisition and analysis. If blinding was not possible, describe why OR explain why blinding was not relevant to your study.

Did the study involve field work? ☐ Yes ☐ No

Field work, collection and transport

Field conditions	Describe the study conditions for field work, providing relevant parameters (e.g. temperature, rainfall).
Location	State the location of the sampling or experiment, providing relevant parameters (e.g. latitude and longitude, elevation, water depth).
Access & import/export	Describe the efforts you have made to access habitats and to collect and import/export your samples in a responsible manner and in compliance with local, national and international laws, noting any permits that were obtained (give the name of the issuing authority, the date of issue, and any identifying information).
Disturbance	Describe any disturbance caused by the study and how it was minimized.

Reporting for specific materials, systems and methods

We require information from authors about some types of materials, experimental systems and methods used in many studies. Here, indicate whether each material, system or method listed is relevant to your study. If you are not sure if a list item applies to your research, read the appropriate section before selecting a response.

Materials & experimental systems

n/a	Involved in the study	
☒	☐	Antibodies
☒	☐	Eukaryotic cell lines
☒	☐	Palaeontology and archaeology
☒	☐	Animals and other organisms
☒	☐	Clinical data
☒	☐	Dual use research of concern
☒	☐	Plants

Methods

n/a	Involved in the study	
☒	☐	ChIP-seq
☒	☐	Flow cytometry
☐	☒	MRI-based neuroimaging

Antibodies

Antibodies used	Describe all antibodies used in the study; as applicable, provide supplier name, catalog number, clone name, and lot number.
Validation	Describe the validation of each primary antibody for the species and application, noting any validation statements on the manufacturer's website, relevant citations, antibody profiles in online databases, or data provided in the manuscript.

Eukaryotic cell lines

Policy information about cell lines and Sex and Gender in Research

Cell line source(s)	*State the source of each cell line used and the sex of all primary cell lines and cells derived from human participants or vertebrate models.*
Authentication	*Describe the authentication procedures for each cell line used OR declare that none of the cell lines used were authenticated.*
Mycoplasma contamination	*Confirm that all cell lines tested negative for mycoplasma contamination OR describe the results of the testing for mycoplasma contamination OR declare that the cell lines were not tested for mycoplasma contamination.*
Commonly misidentified lines (See ICLAC register)	*Name any commonly misidentified cell lines used in the study and provide a rationale for their use.*

Palaeontology and Archaeology

Specimen provenance	*Provide provenance information for specimens and describe permits that were obtained for the work (including the name of the issuing authority, the date of issue, and any identifying information). Permits should encompass collection and, where applicable, export.*
Specimen deposition	*Indicate where the specimens have been deposited to permit free access by other researchers.*
Dating methods	*If new dates are provided, describe how they were obtained (e.g. collection, storage, sample pretreatment and measurement), where they were obtained (i.e. lab name), the calibration program and the protocol for quality assurance OR state that no new dates are provided.*

☐ Tick this box to confirm that the raw and calibrated dates are available in the paper or in Supplementary Information.

Ethics oversight	*Identify the organization(s) that approved or provided guidance on the study protocol, OR state that no ethical approval or guidance was required and explain why not.*

Note that full information on the approval of the study protocol must also be provided in the manuscript.

Animals and other research organisms

Policy information about studies involving animals; ARRIVE guidelines recommended for reporting animal research, and Sex and Gender in Research

Laboratory animals	*For laboratory animals, report species, strain and age OR state that the study did not involve laboratory animals.*
Wild animals	*Provide details on animals observed in or captured in the field; report species and age where possible. Describe how animals were caught and transported and what happened to captive animals after the study (if killed, explain why and describe method; if released, say where and when) OR state that the study did not involve wild animals.*
Reporting on sex	*Indicate if findings apply to only one sex; describe whether sex was considered in study design, methods used for assigning sex. Provide data disaggregated for sex where this information has been collected in the source data as appropriate; provide overall numbers in this Reporting Summary. Please state if this information has not been collected. Report sex-based analyses where performed, justify reasons for lack of sex-based analysis.*
Field-collected samples	*For laboratory work with field-collected samples, describe all relevant parameters such as housing, maintenance, temperature, photoperiod and end-of-experiment protocol OR state that the study did not involve samples collected from the field.*
Ethics oversight	*Identify the organization(s) that approved or provided guidance on the study protocol, OR state that no ethical approval or guidance was required and explain why not.*

Note that full information on the approval of the study protocol must also be provided in the manuscript.

Clinical data

Policy information about clinical studies

All manuscripts should comply with the ICMJE guidelines for publication of clinical research and a completed CONSORT checklist must be included with all submissions.

Clinical trial registration	*Provide the trial registration number from ClinicalTrials.gov or an equivalent agency.*
Study protocol	*Note where the full trial protocol can be accessed OR if not available, explain why.*
Data collection	*Describe the settings and locales of data collection, noting the time periods of recruitment and data collection.*
Outcomes	*Describe how you pre-defined primary and secondary outcome measures and how you assessed these measures.*

Dual use research of concern

Policy information about dual use research of concern

Hazards

Could the accidental, deliberate or reckless misuse of agents or technologies generated in the work, or the application of information presented in the manuscript, pose a threat to:

No	Yes	
☐	☐	Public health
☐	☐	National security
☐	☐	Crops and/or livestock
☐	☐	Ecosystems
☐	☐	Any other significant area

Experiments of concern

Does the work involve any of these experiments of concern:

No	Yes	
☐	☐	Demonstrate how to render a vaccine ineffective
☐	☐	Confer resistance to therapeutically useful antibiotics or antiviral agents
☐	☐	Enhance the virulence of a pathogen or render a nonpathogen virulent
☐	☐	Increase transmissibility of a pathogen
☐	☐	Alter the host range of a pathogen
☐	☐	Enable evasion of diagnostic/detection modalities
☐	☐	Enable the weaponization of a biological agent or toxin
☐	☐	Any other potentially harmful combination of experiments and agents

Plants

Seed stocks	*Report on the source of all seed stocks or other plant material used. If applicable, state the seed stock centre and catalogue number. If plant specimens were collected from the field, describe the collection location, date and sampling procedures.*
Novel plant genotypes	*Describe the methods by which all novel plant genotypes were produced. This includes those generated by transgenic approaches, gene editing, chemical/radiation-based mutagenesis and hybridization. For transgenic lines, describe the transformation method, the number of independent lines analyzed and the generation upon which experiments were performed. For gene-edited lines, describe the editor used, the endogenous sequence targeted for editing, the targeting guide RNA sequence (if applicable) and how the editor was applied.*
Authentication	*Describe any authentication procedures for each seed stock used or novel genotype generated. Describe any experiments used to assess the effect of a mutation and, where applicable, how potential secondary effects (e.g. second site T-DNA insertions, mosiacism, off-target gene editing) were examined.*

ChIP-seq

Data deposition

☐ Confirm that both raw and final processed data have been deposited in a public database such as GEO.

☐ Confirm that you have deposited or provided access to graph files (e.g. BED files) for the called peaks.

Data access links<br>*May remain private before publication.*	*For "Initial submission" or "Revised version" documents, provide reviewer access links. For your "Final submission" document, provide a link to the deposited data.*
Files in database submission	*Provide a list of all files available in the database submission.*
Genome browser session<br>(e.g. UCSC)	*Provide a link to an anonymized genome browser session for "Initial submission" and "Revised version" documents only, to enable peer review. Write "no longer applicable" for "Final submission" documents.*

Methodology

Replicates	*Describe the experimental replicates, specifying number, type and replicate agreement.*

Sequencing depth	*Describe the sequencing depth for each experiment, providing the total number of reads, uniquely mapped reads, length of reads and whether they were paired- or single-end.*
Antibodies	*Describe the antibodies used for the ChIP-seq experiments; as applicable, provide supplier name, catalog number, clone name, and lot number.*
Peak calling parameters	*Specify the command line program and parameters used for read mapping and peak calling, including the ChIP, control and index files used.*
Data quality	*Describe the methods used to ensure data quality in full detail, including how many peaks are at FDR 5% and above 5-fold enrichment.*
Software	*Describe the software used to collect and analyze the ChIP-seq data. For custom code that has been deposited into a community repository, provide accession details.*

Flow Cytometry

Plots

Confirm that:

- [ ] The axis labels state the marker and fluorochrome used (e.g. CD4-FITC).
- [ ] The axis scales are clearly visible. Include numbers along axes only for bottom left plot of group (a 'group' is an analysis of identical markers).
- [ ] All plots are contour plots with outliers or pseudocolor plots.
- [ ] A numerical value for number of cells or percentage (with statistics) is provided.

Methodology

Sample preparation	*Describe the sample preparation, detailing the biological source of the cells and any tissue processing steps used.*
Instrument	*Identify the instrument used for data collection, specifying make and model number.*
Software	*Describe the software used to collect and analyze the flow cytometry data. For custom code that has been deposited into a community repository, provide accession details.*
Cell population abundance	*Describe the abundance of the relevant cell populations within post-sort fractions, providing details on the purity of the samples and how it was determined.*
Gating strategy	*Describe the gating strategy used for all relevant experiments, specifying the preliminary FSC/SSC gates of the starting cell population, indicating where boundaries between "positive" and "negative" staining cell populations are defined.*

- [ ] Tick this box to confirm that a figure exemplifying the gating strategy is provided in the Supplementary Information.

Magnetic resonance imaging

Experimental design

Design type	To assess enduring brain effects, participants attended three scanning sessions, 1-month apart: scan 1 collected baseline data, scan 2 served as the one-month follow-up for 1 mg control as well as a baseline for post-25mg interventions, and scan 3 served as the one-month follow-up for 25 mg psilocybin and was the primary study endpoint.<br><br>Scans included structural diffusion-weighted MRI as well as resting-state (seed-based rsFC; brain network modularity) and block-design (emotional faces paradigm) functional MRI.
Design specifications	Seed-based resting state functional connectivity (RSFC).<br>Based on prior hypotheses, four seeds were chosen for these analyses: (i) bilateral parahippocampus (PH), (ii) bilateral amygdala, (iii) ventromedial prefrontal cortex (vmPFC), and (iv) subgenual anterior cingulate cortex (sgACC). The PH seed was constructed by combining the anterior and posterior parahippocampal gyrus from the Harvard-Oxford probabilistic atlas, which was then thresholded at 50%. The vmPFC seed was the same as one previously used by our team in analyses of the acute effects of LSD, psilocybin and MDMA4. The sgACC seed was a 5 mm sphere centered at MNI coordinates ±2 28 -5. Bilateral amygdala seed was based on Harvard-Oxford probabilistic atlas, threshold at 50%. Mean time-series were derived for these seeds for each resting-state scan. The RSFC analyses were performed using FSL's FEAT for each subject. Pre-whitening (FILM) was applied. A higher-level analysis was performed to compare pre-treatment (scan 2) and post-treatment (scan 3) conditions using a mixed-effects GLM (FLAME 1 + 2), cluster corrected ($Z>2.3$, $p<0.05$). MRIcron was used to display the results.<br><br>Emotional faces paradigm procedures.<br>The emotional faces paradigm was a block-design task lasting 8 min. Participants used a mirror mounted on the head-coil to view a screen mounted in the rear of the scanner bore, where visual stimuli were back-projected through a wave-guide in the rear wall of the scanner room. Participants were shown faces with either fearful, happy, or neutral expressions, selected from the Karolinska Directed Emotional Faces set13. An equal number of male and female faces

were selected for the task. Each face was presented on screen for 3 s, and five faces of the same expression were presented in each 15 s block. Rest blocks (also 15 s) were also included, and there were 8 repetitions of each block type, presented in a pseudo-random sequence (32 blocks in total). Three versions of the task were used and the order of the task versions on each scanning visit was counter-balanced across participants. Participants passively viewed the faces but were instructed to press a single button with their thumb with the presentation of each new face, to confirm that they were paying attention to the stimuli.

For the emotional faces paradigm, we used the same resting-state preprocessing pipeline outlined above but with one modification – as in our previous emotional faces research. The scrubbing threshold was increased to 0.9, as this is a threshold that better suits task paradigms15. No subjects were excluded due to head motion, and 25 subjects were used for the final analysis. The mean percentage of volumes scrubbed for scan 1, scan 2, and scan 3 was 1.1 ± 1.8%, 1.1 ± 2.3%, and 1.1 ± 1.9, respectively.

Different approaches were used to investigate changes in amygdala response in this paradigm: (i) voxelwise analysis within a bilateral amygdala mask (Harvard-Oxford atlas, probability >50%); (ii) calculating mean amygdala signal of left and right amygdala ROIs; and (iii) A whole-brain voxelwise analysis. For all approaches, a standard GLM was used for the first analysis step, as implemented in the FEAT module in FSL. Regressors derived from the onset times of each stimulus condition were convolved with a Gamma function in order to simulate the Haemodynamic Response Function (HRF). Prewhitening (FILM) was applied to correct for autocorrelations. Contrasts were defined that isolated activity related to each stimulus condition (fearful, happy, neutral) relative to the baseline, and comparisons were also made that contrasted between stimulus conditions, as appropriate i.e., [fearful > happy+neutral] and [happy > fearful+neutral]. Mixed-effects GLM (FLAME-1+2) was used for the voxelwise analysis with a statistical threshold of Z>2.3, (cluster-corrected for multiple comparisons, p<0.050).

Brain network modularity.

Brain network modularity was computed on cortical (200 x 200)16 interregional RSFC. As summarized by the Q value, modularity is a measure of the decomposability of brain connectivity into distinct modules, where each module represents a set of brain regions that exhibit strong RSFC with each other (intra-modular RSFC) and weaker RSFC with regions of other modules (inter-modular RSFC).

For the modularity analyses, the RS-fMRI timeseries data was parcellated into 200 regions based on the Schaefer local-global parcellation and the Pearson's (r) correlation was computed between regional timeseries to create a 200 x 200 RSFC matrix. Modularity was then computed on this (unthresholded) matrix using the Louvain algorithm implemented with the Network Community Toolbox (http://commdetect.weebly.com/), treating negative values asymmetrically. This algorithm finds modular partitions of the network (i.e., RSFC matrix) which optimize the modularity value, Q, by grouping nodes into non-overlapping modules (sets of regions) that maximize intra-modular and minimize inter-modular connections. The Louvain algorithm was run iteratively 100 times at the individual-subject level and the average Q value across these runs was used for each subject. Given that modularity is sensitive to the average correlation strength of the network, Q values were normalized for each subject by the mean Q value generated by 100 randomly shuffled ('rewired') permutations of that subject's RSFC matrix.

Structural MRI procedures.

Diffusion-weighted MRI (dMRI) data was acquired with the following acquisition parameters: 64 directions with b = 1000 s/mm2; 6 images without diffusion-weighting; 1 image without diffusion-weighting and with the phase encoding direction reversed; TE = 88 ms; TR = 3010 ms; voxel size = 1.9 x 1.9 x 2.0 mm3; 72 slices. The acquisition was repeated three times to maximize signal-to-noise ratio (SNR). The images were preprocessed using MRtrix3 and the following steps: random matrix denoising, Gibbs-ringing reduction, distortion and motion correction, and bias field correction.

Region of interest (ROI) analysis was chosen for studying white matter plasticity. TractSeg's pre-trained neural network was used to segment white matter and its outputs, representing probabilities of the tract being present in a voxel, were converted into binary masks with a threshold of 0.975 for reliable and reproducible segmentation. The following association fibers were included in the analysis: arcuate fascicle, cingulum, inferior occipito-frontal fascicle, inferior longitudinal fascicle, middle longitudinal fascicle, superior longitudinal fascicle, and uncinate fascicle. Furthermore, the following tracts were included in the analysis because of the results of the functional MRI results showing changes in brain activity in the frontal lobe: anterior thalamic radiation, rostrum of the corpus callosum, genu of the corpus callosum, striato-prefontal tract (PFC-STR), and thalamo-prefrontal tract (PFC-THA). ROIs from the different hemispheres were merged.

The diffusion tensor was estimated in each voxel using an iterated weighted least squares algorithm in Mrtrix319,26,27. The mean values of axial diffusivity (AD) and radial diffusivity (RD) were calculated in each ROI for each subject and time point. Statistical analysis was performed on AD and RD that measure diffusion along orthogonal directions and are independent. In addition, diffusion tensors were estimated with a free-water component, done to remove partial volume effects28. Repeated measures ANOVA was performed on AD and RD separately for each tract with Bonferroni multiple comparisons correction. The analysis was then repeated for mean diffusivity (MD) and fractional anisotropy (FA).

Behavioral performance measures

Cognitive flexibility.

Cognitive flexibility was measured via an aesthetically modified version of the intra-dimensional/extra-dimensional (IDED) task present in the CANTAB neuropsychological test automated battery29, which was kindly provided by Adam Hampshire, C3NL, Imperial College London. The IDED task consists of 9 stages that require simple discrimination learning, compound discrimination learning, abstraction, attentional set-shifting, and reversal learning. Two stimuli are presented to the participant at a time. At any given phase, a hidden rule determines the correct response to the trial. Participants are required to determine the rule and chose the correct stimuli in 6 consecutive trials before progressing

to the next phase. Participants are not informed when the rule changes, only receiving ticks or crosses as feedback for correct or incorrect responses, respectively. Participants fail the task upon responding incorrectly 30 times within any given phase. Stimuli sets are pseudorandomized (different sets of lines and shapes from a given pool) between timepoints. Errors made at each phase were adjusted to the total number of stimuli presented within each phase, representing a measure of 'phase-accuracy' in order to assess changes in IDED performance across time-points while controlling for individual variability in learning rates. Participants completed the IDED task 1-day before and one-month after each dosing day.

Insight.
Psychological insight was measured via the Psychological Insight Scale (PIS)30. The PIS is a 6-7 item questionnaire that measures psychological insightfulness following a psychedelic experience (PIS-6) and accompanied behavioural changes (PIS-7). The PIS is scored using a VAS (0–100, with incremental units of one) with zero defined as 'no more than usual' and 100 defined as 'much more than usual'. Participants completed the PIS at 1-day, 2-weeks and one-month after each dosing day.

Well-being.
Psychological well-being was measured using the 14-item Well-being Warwick-Edinburgh Mental Wellbeing Scale (WEMWBS)31. The WEMWBS is designed to assess mental well-being itself and not the determinants of mental well-being – e.g. resilience, problem solving, etc. The WEMWBS includes hedonic (i.e. happiness, life satisfaction) and eudaimonic (i.e. positive relationships, psychological functioning) items which together measure mental well-being. Items are rated on a 5-point Likert scale (from 1=none of the time to 5=all of the time) to yield a total summed score, with a minimum possible score of 14 and a maximum score of 70 (population norms: M=51, SD=9)32. Participants completed the WEMWBS 1-day before and at 2-weeks and 4-weeks after each dosing day.

Persisting effects questionnaire.
Inspired by previous methods by Griffiths et al33, six months post-dosing, participants reflected on the peak of each dosing session and answered the following three questions: 1) "How profound was the state of consciousness you experienced?", 2) "How intense was the state of consciousness you experienced?", and 3) "How unusual was the state of consciousness you experienced?" (See Figure S16). Each item was rated using an eight-point Likert scale, from 1 = "no more 'profound / intense / unusual' than routine everyday states of consciousness" to 8 = "the single most 'profound / intense / unusual' state of consciousness of my life, that I can recall").

Acquisition

Imaging type(s)

Functional MRI (seed-based rsFC, network modularity and block design); diffusion MRI.

Field strength

Imaging was performed on a 3T Siemens Tim Trio using a 12-channel head coil.

Sequence & imaging parameters

T2*-weighted echo-planar images (EPI) were acquired using the MB2R2 protocol3 with interleaved slice acquisitions for the functional scans using 3 mm isotropic voxels, TR = 1250 ms, TE = 30 ms, 44 axial slices, 192 mm in-plane FOV, flip angle = 80°, bandwidth = 2232 Hz/pixel, GRAPPA acceleration = 2, number of volumes = 384. Both the resting state and emotional faces paradigm scans were each 8 minutes in duration. Resting-state scans were completed with eyes-closed.

Area of acquisition

whole brain

Diffusion MRI ☒ Used ☐ Not used

Preprocessing

Preprocessing software

dMRI: images were preprocessed using MRtrix3 and the following steps: random matrix denoising, Gibbs-ringing reduction, distortion and motion correction, and bias field correction.

fMRI: FMRIB Software Library (FSL)6, Analysis of Functional NeuroImages (AFNI)7, Freesurfer8 and Advanced Normalization Tools (ANTS)9 were used to analyze the resting-state data. Motion was measured using frame-wise displacement (FD)10. The following preprocessing stages were performed: 1) removal of the first three volumes; 2) de-spiking (3dDespike, AFNI); 3) motion correction (3dvolreg, AFNI) by registering each volume to the volume most similar, in the least squares sense, to all others (in-house code); 4) brain extraction (BET, FSL); 5) rigid body registration to anatomical scans (FSL, BBR); 6) non-linear registration to 2mm MNI brain (Symmetric Normalization (SyN), ANTS); 7) scrubbing11 using an FD threshold of 0.4 (the mean percentage of volumes scrubbed for scan 1, scan 2, and scan 3 was 0.9 ± 1.6%, 0.9 ± 1.4%, and 2.6 ± 5.3, respectively). Scrubbed volumes were replaced with the mean of the surrounding volumes. Additional preprocessing steps included: 8) spatial smoothing (FWHM) of 6mm (3dBlurInMask, AFNI); 9) band-pass filtering between 0.01 to 0.08 Hz (3dFourier, AFNI); 10) linear and quadratic de-trending (3dDetrend, AFNI); 11) regressing out 9 nuisance regressors (the same bandpass filter was applied on the nuisance regressors): out of these, 6 were motion-related (3 translations, 3 rotations) and 3 were anatomically-related (not smoothed). Specifically, the anatomical nuisance regressors were: 1) ventricles (Freesurfer, eroded in 2mm space), 2) draining veins (DV) (FSL's CSF minus Freesurfer's Ventricles, eroded in 1mm space) and 3) local white matter (WM) (FSL's WM minus Freesurfer's subcortical grey matter (GM) structures, eroded in 2mm space). Regarding local WM regression12, AFNI's 3dLocalstat was used to calculate the mean local WM time-series for each voxel, using a 25mm radius sphere centered on each voxel.

Normalization

Non-linear registration to 2mm MNI brain (Symmetric Normalization (SyN), ANTS)

Normalization template

See above.

Noise and artifact removal

The criterion for exclusion was subjects with > 20% scrubbed volumes when the scrubbing threshold is FD = 0.4. Two subjects

were excluded to high levels of head motion.

Volume censoring: > 20% scrubbed volumes when the scrubbing threshold is FD = 0.4

Statistical modeling & inference

Model type and settings: see above

Effect(s) tested:

dMRI: The diffusion tensor was estimated in each voxel using an iterated weighted least squares algorithm in Mrtrix319,26,27. The mean values of axial diffusivity (AD) and radial diffusivity (RD) were calculated in each ROI for each subject and time point. Statistical analysis was performed on AD and RD that measure diffusion along orthogonal directions and are independent. In addition, diffusion tensors were estimated with a free-water component, done to remove partial volume effects28. Repeated measures ANOVA was performed on AD and RD separately for each tract with Bonferroni multiple comparisons correction. The analysis was then repeated for mean diffusivity (MD) and fractional anisotropy (FA).

Different approaches were used to investigate changes in amygdala response in this paradigm: (i) voxelwise analysis within a bilateral amygdala mask (Harvard-Oxford atlas, probability >50%); (ii) calculating mean amygdala signal of left and right amygdala ROIs; and (iii) A whole-brain voxelwise analysis. For all approaches, a standard GLM was used for the first analysis step, as implemented in the FEAT module in FSL. Regressors derived from the onset times of each stimulus condition were convolved with a Gamma function in order to simulate the Haemodynamic Response Function (HRF). Prewhitening (FILM) was applied to correct for autocorrelations. Contrasts were defined that isolated activity related to each stimulus condition (fearful, happy, neutral) relative to the baseline, and comparisons were also made that contrasted between stimulus conditions, as appropriate i.e., [fearful > happy+neutral] and [happy > fearful+neutral]. Mixed-effects GLM (FLAME-1+2) was used for the voxelwise analysis with a statistical threshold of Z>2.3, (cluster-corrected for multiple comparisons, p<0.050).

Mean time-series were derived for the seeds for each resting-state scan. The RSFC analyses were performed using FSL's FEAT for each subject. Pre-whitening (FILM) was applied. A higher-level analysis was performed to compare pre-treatment (scan 2) and post-treatment (scan 3) conditions using a mixed-effects GLM (FLAME 1 + 2), cluster corrected (Z>2.3, p<0.05).

For the modularity analyses, the RS-fMRI timeseries data was parcellated into 200 regions based on the Schaefer local-global parcellation16 and the Pearson's (r) correlation was computed between regional timeseries to create a 200 x 200 RSFC matrix. Modularity was then computed on this (unthresholded) matrix using the Louvain algorithm17 implemented with the Network Community Toolbox (http://commdetect.weebly.com/), treating negative values asymmetrically. This algorithm finds modular partitions of the network (i.e., RSFC matrix) which optimize the modularity value, Q, by grouping nodes into non-overlapping modules (sets of regions) that maximize intra-modular and minimize inter-modular connections18

Specify type of analysis: ☐ Whole brain ☐ ROI-based ☒ Both

Statistic type for inference: see above

(See Eklund et al. 2016)

Correction: see above

Models & analysis

n/a	Involved in the study
☐	☒ Functional and/or effective connectivity
☐	☒ Graph analysis
☒	☐ Multivariate modeling or predictive analysis

Functional and/or effective connectivity: see above

Graph analysis: see above

Multivariate modeling and predictive analysis: *Specify independent variables, features extraction and dimension reduction, model, training and evaluation metrics.*
